# Pre-operative stress testing in the evaluation of patients undergoing non-cardiac surgery: A systematic review and meta-analysis

**DOI:** 10.1371/journal.pone.0219145

**Published:** 2019-07-11

**Authors:** Bindu Kalesan, Heidi Nicewarner, Sunny Intwala, Christopher Leung, Gary J. Balady

**Affiliations:** 1 Department of Medicine and Community Health Sciences, Boston University School of Medicine and Public Health, Boston, Massachusetts, United States of America; 2 Department of Medicine, Boston Medical Center, Boston University Medical Campus, Boston, Massachusetts, United States of America; Vita Salute University of Milan, ITALY

## Abstract

**Background:**

Pre-operative stress testing is widely used to evaluate patients for non-cardiac surgeries. However, its value in predicting peri-operative mortality is uncertain. The objective of this study is to assess the type and quality of available evidence in a comprehensive and statistically rigorous evaluation regarding the effectiveness of pre-operative stress testing in reducing 30-day post -operative mortality following non -cardiac surgery.

**Methods:**

The databases of MEDLINE, EMBASE, and CENTRAL databases (from inception to January 27, 2016) were searched for all studies in English. We included studies with pre-operative stress testing prior to 10 different non-cardiac surgery among adults and excluded studies with sample size<15. The data on study characteristics, methodology and outcomes were extracted independently by two observers and checked by two other observers. The primary outcome was 30-day mortality. We performed random effects meta-analysis to estimate relative risk (RR) and 95% confidence intervals (95% CI) in two-group comparison and pooled the rates for stress test alone. Heterogeneity was assessed using *I*^*2*^ and methodological quality of studies using Newcastle-Ottawa Quality Assessment Scale. The predefined protocol was registered in PROSPERO #CRD42016049212.

**Results:**

From 1807 abstracts, 79 studies were eligible (297,534 patients): 40 had information on 30-day mortality, of which 6 studies compared stress test versus no stress test. The risk of 30-day mortality was not significant in the comparison of stress testing versus none (RR: 0.79, 95% CI = 0.35–1.80) along with weak evidence for heterogeneity. For the studies that evaluated stress testing without a comparison group, the pooled rates are 1.98% (95% CI = 1.25–2.85) with a high heterogeneity. There was evidence of potential publication bias and small study effects.

**Conclusions:**

Despite substantial interest and research over the past 40 years to predict 30-day mortality risk among patients undergoing non-cardiac surgery, the current body of evidence is insufficient to derive a definitive conclusion as to whether stress testing leads to reduced peri-operative mortality.

## Introduction

More than 312 million major surgical procedures are performed worldwide each year.[1] Of these, approximately five million major non-cardiac surgeries occur in the United States[2] [3] It is estimated that non-cardiac surgery has a complication rate as high as 11%, and 42% of these complications are cardiovascular in nature.[3] Within 30-days after non-cardiac surgery, cardiovascular complications are the leading cause of peri-operative death,[2] with a rate currently estimated at 1.7% in the United States,[2] that is similar worldwide.[3] Although the occurrence of peri-operative adverse cardiovascular events has declined over the past decade,[2] the continued growth in the aged population along with its associated co-morbidities presents a burgeoning challenge to mitigate surgical risk in these progressively complicated patients.

Comprehensive consideration of the type of surgery that is to be performed along with careful and appropriate evaluation of each specific patient aims to yield a benefit-risk assessment that guides subsequent decisions and patient management. Stress testing is among the most fundamental and widely used tools in the evaluation of patients with cardiovascular disease (CVD). Despite more than four decades of research, very few randomized trials have addressed the value of pre-operative stress testing.

Continued advances in surgical techniques and peri-operative monitoring have counterbalanced changes in patient demographics in those undergoing non-cardiac surgeries. Indiscriminate routine stress testing may lead to further unnecessary downstream testing, including additional medical treatments, costly invasive procedures that may delay the planned surgical procedure or possibly increase perioperative adverse event rates that provide no benefit to the patient.[4]

Therefore, the objective of this study is to assess the type and quality of available evidence in a comprehensive and statistically rigorous evaluation regarding the effectiveness of pre-operative stress testing in reducing 30-day post -operative mortality following non -cardiac surgery. We hypothesized that the current evidence is not adequate for setting guidelines due to lack of studies of sufficient quality. We also addressed the following secondary questions: 1) does pre-operative stress testing change relative to the era in which the surgery was performed, 2) does the benefit of reduction in mortality due to pre-operative stress testing differ relative to the types of surgery performed, 3) are there differences in the benefit of reduction in mortality related to pre-operative testing relative to the type of test that was performed, and 4) are there variables derived from pre-operative stress testing that predict 30 day post-operative mortality or other cardiovascular events including non-fatal myocardial infarction, heart failure, or stroke.

## Materials and methods

This pooled data analysis of aggregate data from published studies was performed according to a predefined protocol in PROSPERO #CRD42016049212. Ethics committee approval was not required since only aggregate data was used from published literature.

### Search strategy and selection criteria

We performed a systematic literature search in MEDLINE, EMBASE, and CENTRAL from inception to January 27, 2016, of all studies with non-cardiac surgery and stress testing in humans. The search criteria in three databases are presented in S1 Appendix. The search yielded 1807 studies. All abstracts were obtained and screened using inclusion and exclusion criteria presented in S2 Appendix. Only studies in English, and among adults were included from 10 different non-cardiac surgical procedures of peripheral vascular, thoracic, abdominal, gynecological, urological, renal transplant, liver transplant, orthopedic, gastric bypass, abdominal aortic aneurysm procedures. The types of stress tests used were exercise tolerance, pharmacological nuclear, adenosine nuclear, Persantine nuclear, dobutamine nuclear, exercise nuclear, exercise echocardiogram, dobutamine echocardiogram, cardiopulmonary, metabolic, stress test with gas exchange analysis and six-minute walk test. We did not apply restrictions based on outcomes. Studies with a sample size of <15 were excluded. An updated search was performed on June 4, 2019 and assessment of new abstracts revealed no new observational studies or clinical trials after our search date. Therefore, we did not update our search.

### Data extraction

**Fig 1:**
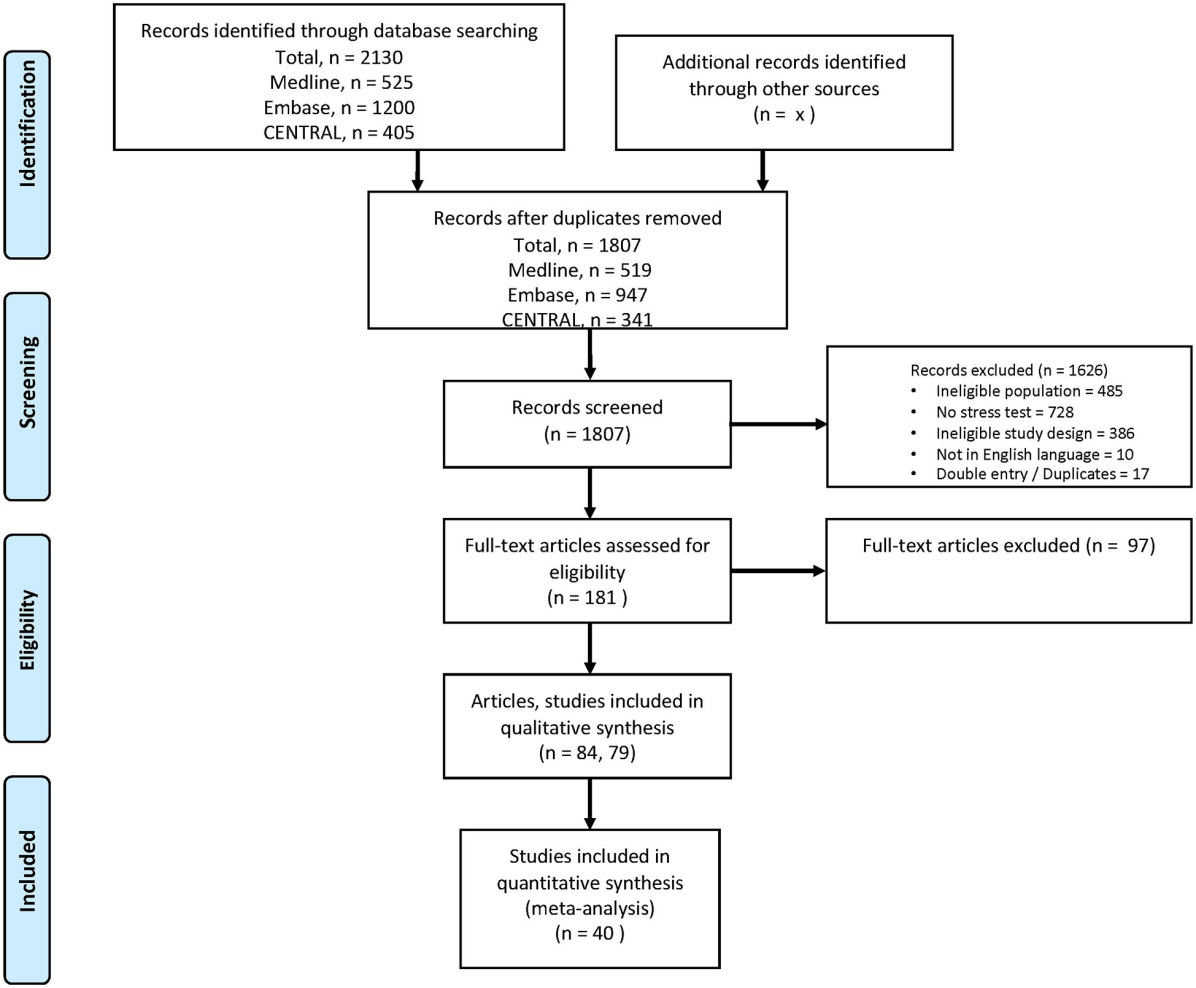
Flow chart. *Adapted from*: Moher D, Liberati A, Tetzlaff J, Altman DG, The PRISMA Group (2009). *P*referred *R*eporting *I*terns for *S*ystematic Reviews and *M*eta-*A*nalyses: The PRISMA Statement. PLoS Med 6(7): el000097. doi:10.1371/journal.pmedl000097. For more information, visit www.prisma-statement.org.

Two authors (H. N. and S. I.) independently screened the abstracts (S3 Appendix). The concordance between screeners was high-agreement of 90.4% and Kappa of 75.4%. We obtained the full text of 181 articles and further screened the full text using inclusion and exclusion criteria (S4 Appendix). Full texts were further assessed, and 97 articles were excluded for the same eligibility criteria. There were 84 articles remaining (79 distinct studies) that were used for the final analysis.[5–88] The details of the screening are presented in a flowchart presented in Fig 1. We extracted the total number of patients in the cohorts of patients who received a stress test and those patients who did not receive testing prior to non-cardiac surgery. The primary outcome was 30-day mortality and we extracted the number of deaths in patients who received a stress test and those who did not. The other variables we extracted were study characteristics such as demographic, methodologic, design, length of follow up, type of surgery and type of stress testing. For the randomized trials, as components of methodological quality, we assessed concealment of allocation, blinding of investigators adjudicating clinical events, and the inclusion of all randomized individuals in the analysis according to the intention-to-treat principle.[89, 90] To evaluate study quality of cohort studies, we used the individual criteria of the Newcastle-Ottawa Quality Assessment Scale (S5 Appendix).[91] All data, including outcomes data, were extracted by four of the authors: HN., SI, CL, GJB.

### Stratification and sensitivity analysis

The stratification variables were pre-specified and were the type of surgery performed (vascular, lung, abdominal, and other), type of stress tests administered (exercise-electrocardiogram, nuclear stress, echocardiographic stress, cardiopulmonary testing), evidence of ischemia, time of study publication, and the country from where the data originated. Post-hoc exploration by peak exercise work rate or other test-specific measured variables showed insufficient extractable information. Sensitivity analysis was performed using study quality variables extracted using individual criteria of the Newcastle-Ottawa Quality Assessment scale.[91]

### Data analysis

We used a two-step approach in our analyses. In the first step, we selected those studies which had a comparison of stress test and no stress test and had information on 30-day mortality. In the second step, we conducted a meta-analysis on all studies with a single cohort of those patients who received stress testing and had information on 30-day mortality. In the comparison of stress test versus no stress test, we calculated risk ratios (RR) and 95% confidence intervals (95% CI) as measures of treatment effect using the DerSimonian and Laird random-effects model to combine estimates across studies.[92] We determined heterogeneity across studies using the I^2^ statistic and constructed funnel plots.[93–95] Due to very few studies having a comparison group of non-stress test, we compared and presented studies according to methodological biases. In the second step, we analyzed patients who only received stress testing, using the number of 30-day mortality events in each study and the total sample size of the sample that received the stress test. We calculated the pooled estimates (effect size or proportion) after Freeman-Tukey Double Arcsine Transformation[96] to stabilize the variances.[97] This is a procedure specific to binomial data and uses exact methods. We determined heterogeneity across studies using the I^2^ statistic, constructed funnel plots and assessed funnel plot symmetry using regression test.[98] Then, we explored potential sources of heterogeneity among the studies based on study characteristics (stratified analysis) and according to methodological biases (sensitivity analysis) using random effects meta-regression, calculated the corresponding I^2^ statistic for respective strata and p-for-interaction.

## Results

Among 1807 studies identified in our literature search, 79 distinct studies from 84 articles met inclusion criteria (Fig 1). The year of publication was from 1981 to 2015 and the year of enrollment ranged from 1976 to 2010. The final sample consisted of 297,534 participants with mean age ranging from 32 to 75 years, and the percentage of men in the studies ranging from 19 to 100% (Table 1). The follow-up period varied between peri-operative to 168 months. A total of 46,015 patients received some form of stress test. Only 40 studies reported 30-day mortality.

**Table 1:** Clinical characteristics of clinical trials and cohort studies, N = 79.

Author	Design	Year	Surgery type	No. of participants	% male	Mean age	Type of stress test	No. receiving test	No. with CAD	No. with diabetes	No. with ischemia	No. having surgery	Months follow-up	No. receiving test at end of study
Falcone, R.A.	RCT	2003	S1, S4	99	68	65	T0, T4, T7	46	103	26	7	99	12	46
Poldermans, D.	RCT	2006	S1	770	77	68	T0, T1	386	72	85	99	770	36	386
Poldermans, D.	RCT	2007	S1	101	88	70	T1	101	99	33	101	98	12	101
Mondillo, S	RT	2002	S1	188	76	67	T4, T6, T7	61	52	43	52	188	1	188
Cutler, B.S.	cohort	1981	S1	284	70	62	T2	284			50	100	periop	284
Arous, EJ	cohort	1984	S1	808			T2	808			135	89	60	135
Smith, T.P.	cohort	1984	S2	22	19	56	T9	22	7		CPX	22	1	22
Carliner, N.	cohort	1985	S1, S3, S4, S5, S6, S11	200	70	59	T2	200	57		30	198	1	200
McPhail, N.	cohort	1988	S1	101			T2	101			21	100		101
Lette J.	cohort	1989	S1, S4, S5, S6, S7, S9	66	53	59	T4	66	18	14	21	60		60
McPhail, N.	cohort	1989	S1	64	77	66	T0, T2, T4	60			34			31
Boysen,P.G.	cohort	1990	S2	70	88	62	T2	17	0			17	1	17
Joyce, W	cohort	1990	S1	77	68	68	T2	77	42		21	77	1	77
Holley	cohort	1991	S7	189	68	40	T0, T5	141	0	189	41	122	60	189
Holden	cohort	1992	S2	23	81	68	T9, T10	16			0	16	18	16
Reifsnyder, T.	cohort	1992	S1	126	100	65	T4, T5	103	61	26	51	141	4	103
Davila-Roman, V.G.	cohort	1993	S1	98	69	67	T7	98	59	18	23	93	27	
Epstein, S.K.	cohort	1993	S2	42	98	62	T9	42	10		CPX	42	1	42
Kaaja, R	cohort	1993	S1	58	67	59	T2	58	17		14	58	0.5	58
Older P.	cohort	1993	S1, S4	191	55	70	T9	187			CPX	187		187
Poldermans, D.	cohort	1993	S1	136	85	68	T7	136	56	15	35	136	postop	136
Seeger, J.M.	cohort	1994	S1	318		64	T0, T4, T5	146	82	37	70	318	132	146
Poldermans, D.	cohort	1994	S1	106		75	T7	106			40	104	22	106
Poldermans, D.	cohort	1994	S1	187	84	69	T7	187	69	22	56	187	25	187
Bollinger, C.T	cohort	1995	S2	84	68	63	T9	25				25	6	25
Mocini, D	cohort	1995	S1	60	97	67	T4, T6	52	4	16	18	55	12	52
Poldermans, D.	cohort	1995	S1	302	85	67	T7	302	93	33	72	302	In-hospital	302
Donovan, C.L	cohort	1996	S8	190	52	50	T0, T7	165	7	30	11	71	postop	165
Erickson, C.A.	cohort	1996	S1	209	88	67	T0, T4	147	90	31	91	209	60	147
Pellikka, P.A.	cohort	1996	S1, S4, S9, S11	98	78	73	T7	98	28		28	80		98
D’Angelo, A.J.	cohort	1997	S1	113	79	72	T0, T4	20	52	12	7	113	postop	20
Deville, C.	cohort	1997	S1	283	91	68	T0, T2	204	123		21	283	168	204
Kryzhanovski, V.A.	cohort	1997	S8	88	71	54	T0, T4, T5	64	8		8			64
Larsen, K.R.	cohort	1997	S2	97	69	64	T9	97			CPX	97	1	97
Richter Larsen, K.	cohort	1997	S2	97		64	T2	97						97
Van Damme H.	cohort	1997	S1	156	68	66	T4, T7	150	76	28	30	142	postop	150
Poldermans, D.	cohort	1997	S1	316	82	67	T7	316	92	32	84	316	30	316
Nugent	cohort	1998	S1	36	80	71	T9	36	10	2		30	12	30
Won, A	cohort	1998	S1	171	94	69	T0, T1	136	47	15	36	171	60	160
Fleisher, L.A.	cohort	1999	S1	1666			T0, T1	261				1666	12	261
Gauss, A.	cohort	1999	S1, S4	204	79	67	T2	204	90	35	72	185	1	204
Das, M.K.	cohort	2000	S3, S4, S9, S11	530	57	71	T7	530	264	113	214	530	postop	530
Lacroix, H.	cohort	2000	S1	200	95	65	T0, T4, T7			16	154	200	0.25	195
Williams, K.	cohort	2000	S8	121	64	53	T7	121			2	61		121
Farid, I.	cohort	2002	S1, S4, S6, S9, S11	181			T4, T5, T7, T8	181			27	178	12	181
Villani, F	cohort	2003	S2	150	94	57	T9	150	30		CPX	150	1	150
Ali, M	cohort	2004	S7	190	63	43	T0, T2	47	43		9	47	60	47
Epstein, S.K.	cohort	2004	S8	156	59	46	T9	156			CPX	59	25	156
Golzar, JA	cohort	2004	S1	63	62	64	T4, T5	63		10	0	63	12	63
Park, K.W.	cohort	2005	S1	31		72	T0, T4, T5	28	15	17	28	31	periop	28
Win, T	cohort	2005	S2	101	62	68	T0, T9	99			CPX	101	1	99
McMcCullough, P.A.	cohort	2006	S10	109	25	46	T9	109	11	39	CPX	109	1	109
Bai, J.	cohort	2007	S11	1570		68	T4	1351		193	352	1351	1	1351
Schouten, O.	cohort	2007	S1	77	95	73	T7	77	63	11	35	77	1	77
Forshaw, M.J.	cohort	2008	S3	97			T9	78			CPX	78	hospital discharge	78
Gonzalez, CA	cohort	2008	S2	130	69	52	T0, T10	95				130	96	95
Jaroszewski, D	cohort	2008	S2, S3	294	98	62	T0, T2, T4, T5, T7	184	88	65	75	294	1	184
Schouten, O.	cohort	2008	S1	124	92	74	T7	124	107	18	58	124	72	124
Afolabi, B.A.	cohort	2009	S10	157	31	47	T2, T3, T6	78	18	47	25		1	78
Brunelli, A.	cohort	2009	S2	285		67	T9	263	47			263	postop	263
Kasikcioglu, E.	cohort	2009	S2	49	90	61	T9	49	6		CPX	49		49
Matyal R.	cohort	2010	S1	503	65		T4	503	309	264	160	503	hospital stay	503
Snowden, C.P.	cohort	2010	S1, S4	171	70	69	T9	171	35	30	CPX	123	1	171
Wijeysundera, D.N.	cohort	2010	S1, S2, S4, S6, S9	271082	49	68	T0, T1	23991	26562	49323		271082	12	23991
Aalten, J.	cohort	2011	S7	349	56	50	T0, T3, T7	227	103		15	4	1	227
Bub, G.L	cohort	2011	S1	246	82	74	T0, T1	179	208	50	27	246	1	179
Shetty, V.J.	cohort	2011	S9	109	28		T7	109	16	21	12	103	postop	109
Thompson, A.R.	cohort	2011	S1	102	92	75	T9	102	44	9	CPX	63	33	102
Ausania, F	cohort	2012	S4	50	66	64	T0, T9	20	12	14			33	20
Hartley	cohort	2012	S1	415	84		T9	415	179	43		415	3	415
Koh, A.S.	cohort	2012	Not specified	176	59	61	T4, T5	176		67	65	107	3	176
Prentis, J.M.	cohort	2012	S8	182		52	T7, T9	182			CPX, 1 DSE	64	3	182
Prentis, J.M.	cohort	2012	S1	185	87	73	T9	185	83	24	CPX	185	hospital stay	185
James, S	cohort	2014	S1, S4	100	72	68	T0, T9	83	23	14		83	1	83
Prentis, J.M.	cohort	2013	S6	82	59	69	T9	82			CPX	74	hospital stay	82
Grant, SW	cohort	2014	S1	506	83	73	T9	506	227	48	49	506	67	506
Snipelisky, D.	cohort	2014	S8	66	68	59	T7	66	28	30	28	66	60	66
West, M.A.	cohort	2014	S4	136	65	71	T9	136	14	16	CPX	136	1	136
West, M.A.	cohort	2014	S4	95	79	66	T9	95	14	18	CPX	95	12	95
Castleberry, A.W.	cohort	2015	S2	9526	59	32	T10	9526		1674	6MWT	9526	60	9526
Chaikriangkrai, K.	cohort	2015	S2	324	58	57	T10	324	144		6MWT	324	40	324
Marjanski T.	cohort	2015	S2	318	58	63	T0, T10	253			6MWT	318	3	253
Tolchard S.	cohort	2015	S6	105	84	71	T9	105	24	15	CPX	105	3	105
Ulyett, S.	cohort	2015	S4	405	51	66	T0, T9	101		43	CPX	405	77	101

RCT = randomized control trial, RT = randomized trial, no control

**Types of surgery**: S1 = vascular, S2 = lung, S3 = thoracic, S4 = abdominal, S5 = gynecological, S6 = urological, S7 = renal transplant, S8 = liver transplant, S9 = orthopedic, S10 = gastric bypass, S11 = other

**Types of stress test**: T0 = no stress test, T1 = type unspecified, T2 = exercise tolerance test, T3 = nuclear stress test- type unspecified, T4 = pharmacological nuclear test, T5 = exercise nuclear test, T6 = echo stress test- type unspecified, T7 = dobutamine stress echo, T8 = exercise echo stress test, T9 = cardiopulmonary exercise test, T10 = six-minute walk test

6MWT: 6-minute walk test, CPX: cardiopulmonary exercise test

**Fig 2:**
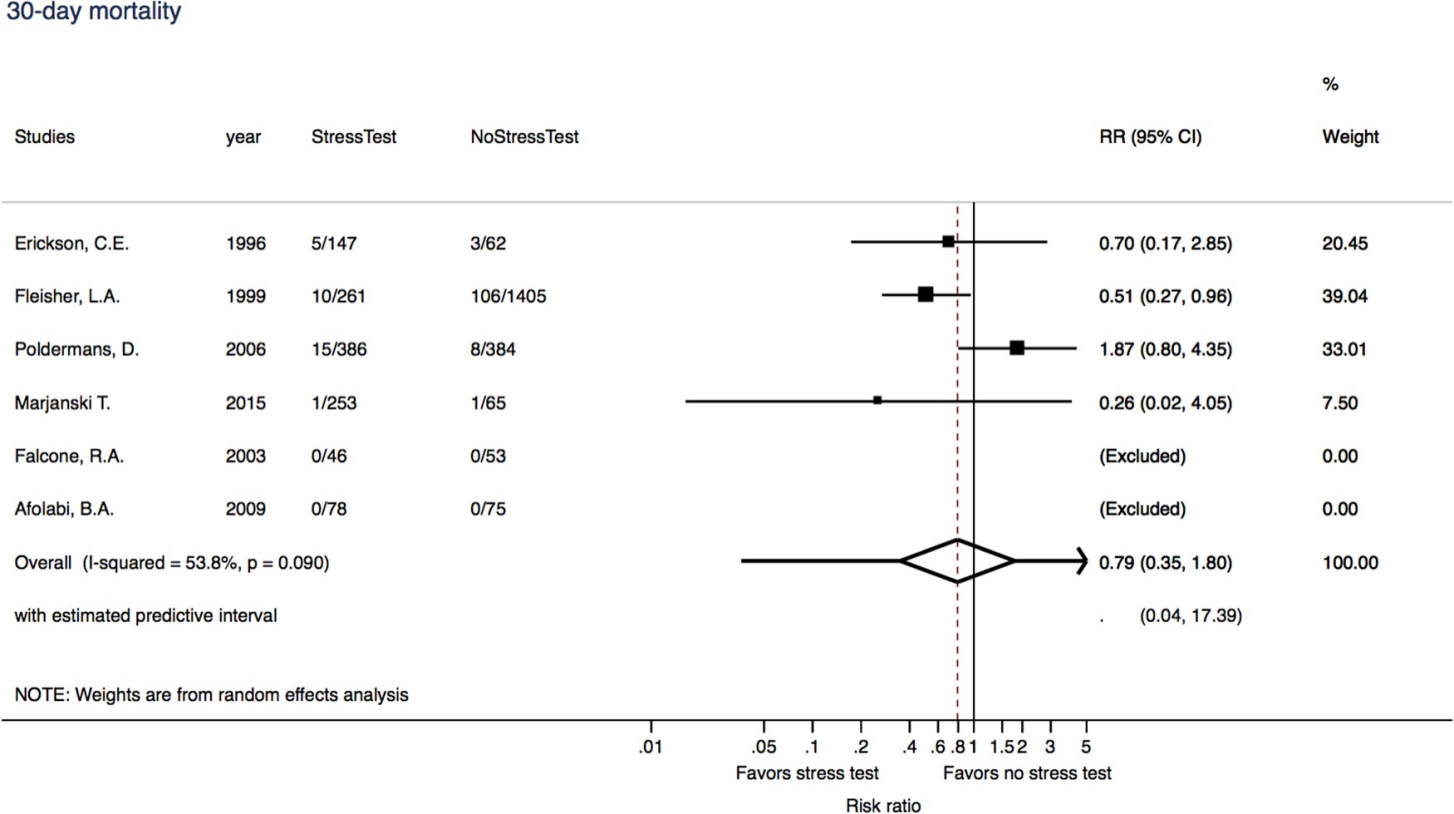
Meta-analysis of 30-day mortality in a comparison of stress test versus no stress test among non-cardiac surgery patients, N = 6 studies. There are only 6 studies which had both groups (had stress test versus no stress test) among 79 studies.

Only six studies had any form of stress test, 30-day mortality and had a comparison of stress test versus non-stress test groups (n = 3219). The range of mean age of patients was 47 to 68 years and the percentage of males was 31% to 88%. Four studies had vascular, one had lung, one abdominal and one gastric bypass surgical procedure. Two studies were randomized clinical trials and the remaining four were cohort studies. Of these, patients in four of these studies had specific surgeries, either vascular, lung, abdominal or gastric bypass procedures. Fig 2 presents the forest plot with RRs of individual studies scattered around the null effect line at one (RR = 0.79 (95% CI = 0.35–1.80). Heterogeneity was high (53.8%), with weak evidence (p = 0.090). The scatter of effect estimates and the prediction line from meta-regression models indicated symmetry, with all studies in the funnel of non-significance at p>0.05 (S3 Fig). The regression test was negative (p = 0.92). In the assessment of biases, both randomized clinical trials were of good methodological quality while the cohort studies had different biases- mainly comparability of the cohorts (S4 Fig).

**Fig 3:**
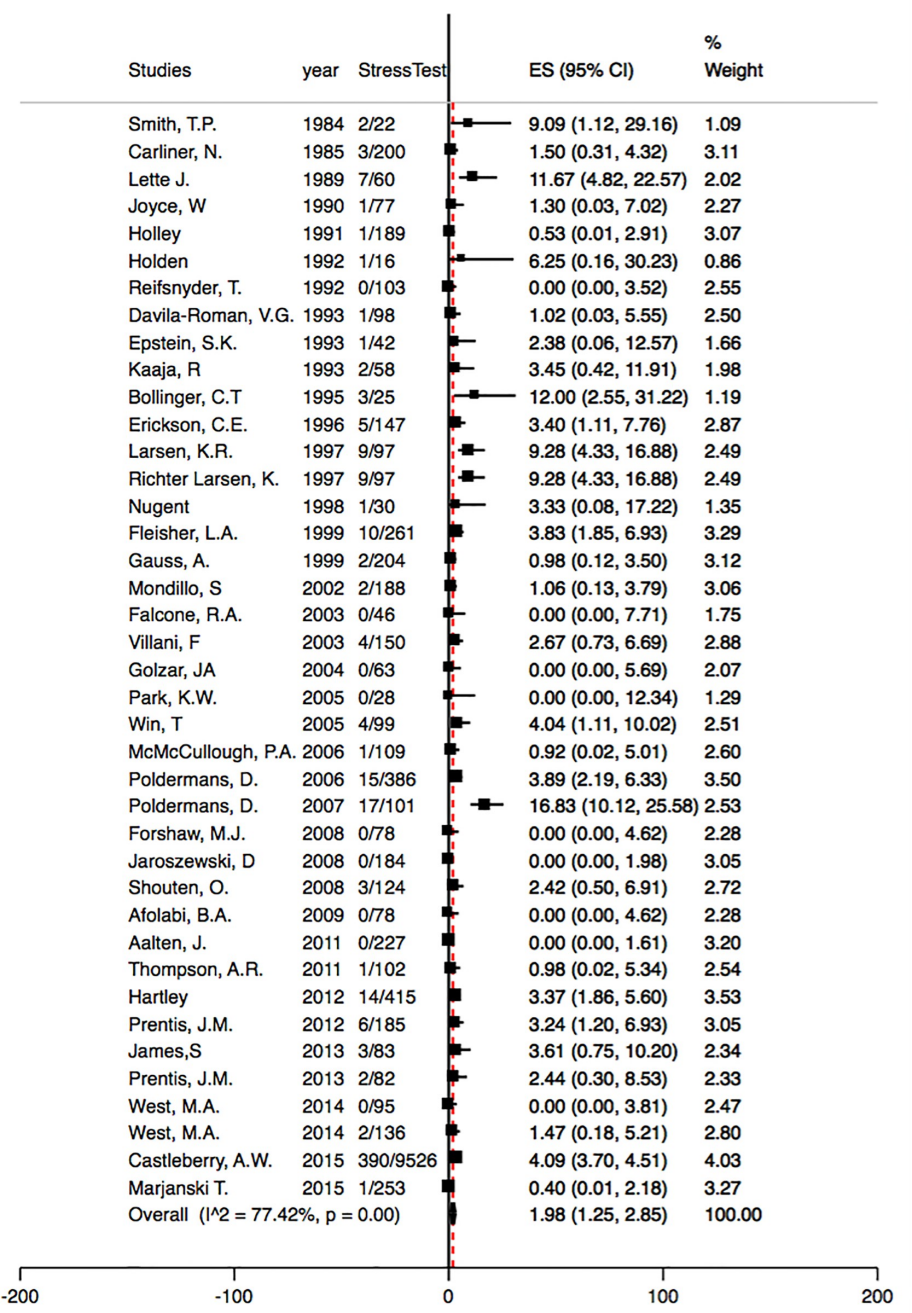
Meta-analysis of 30-day mortality among non-cardiac surgery patients who received stress test using procedures specific to binomial data and exact methods, N = 40. ES is effect size- here it is %. Here we calculate the pooled estimate after Freeman-Tukey Double Arcsine Transformation (Freeman, M. F., and Tukey, J. W. 1950) to stabilize the variances.

Among the 79 studies, only 40 studies had 30-day mortality assessed as an outcome. These 40 studies were published from 1984 to 2015 and a total sample size of 16,886. The range of mean age of patients was from 32 to 75 years and the percentage of males from 19% to 100%. 21 studies had vascular surgery, 11 studies had lung surgery, three had thoracic surgery, seven had abdominal surgery, two had gynecological procedures, two had urological procedures, three had renal transplants, one had orthopedic procedures, and two had gastric bypass surgery. Fig 3 presents the forest plot with proportion (%) of 30-day mortality rates of individual studies scattered around the null effect line at zero. The pooled 30-day mortality rate was 1.98% and 95% CI was 1.25%-2.85%. Heterogeneity was high (77.4%), indicating inconsistencies among studies, and the evidence for heterogeneity was strong (p<0.0001). The results of meta-analysis using standard meta-analysis and using procedures specific to binomial data with continuity correction are presented in S3 and S4 Figs.

Stratified analysis of 30-day mortality among non-cardiac surgery patients who received stress test is presented in Table 2. After excluding three studies with a comparison group, the pooled rate estimates reduced slightly towards the null (1.75%, 95% CI = 1.05%-2.58%) (S5 Fig). Stratified analysis by type of stress test (Table 2) demonstrated no evidence of difference in effect estimates by stress test categories. Cumulative meta-analysis of 30-day mortality is presented in S6 Fig. Stratified analysis by decades of publication year demonstrated highest mortality rate between 1981 to 1989 (5.82%, 95% CI = 0.09%-17.08%) and declined with decades, the pooled estimate during 2010 to 2015 was 1.60% (95% CI = 0.57–3.05) (S7 Fig). There were no differences in pooled estimates by study size (S8 Fig) or types of non-cardiac surgery (Table 2, S9–S19 Figs).

**Table 2:** Stratified analysis of 30-day mortality among non-cardiac surgery patients who received stress test using procedures specific to binomial data and exact methods by study characteristics, N = 40.

	# of studies	# of patients	ES (95% CI), p	I^2^, p-value	P-interaction
All	40	16,886	1.98 (1.25–2.85), <0.0001	77.4, <0.0001	
Comparison group					0.099
No	37	15,916	1.75 (1.05–2.58), <0.0001	75.4, <0.0001	
Yes	3	970	5.11 (0.04–15.8), 0.04	-	
Vascular surgery					0.81
No	19	11,968	1.67 (0.58–3.15), <0.0001	83.6, <0.0001	
Yes	21	4,918	2.36 (1.36–3.58), <0.0001	66.7, <0.0001	
Lung surgery					0.081
No	29	6,132	1.65 (0.89–2.60), <0.0001	69.5, <0.0001	
Yes	11	10,754	3.34 (1.40–2.85), <0.0001	80.9, <0.0001	
Abdominal surgery					0.73
No	33	15,986	2.06 (1.24–3.04), <0.0001	78.2, <0.0001	
Yes	7	900	1.65 (0.29–3.77), <0.0001	65.8, <0.0001	
Other surgery					0.27
No	31	15,343	2.47 (1.64–3.43), <0.0001	70.3, <0.0001	
Yes	9	1,543	0.76 (0.02–2.18), 0.03	70.4, <0.0001	
Stress test type					0.33*
Multiple	12	3849	2.14 (0.38–4.86), <0.0001	84.7, <0.0001	
ETT^a^	4	539	1.30 (0.40–2.57), <0.0001	0.00, 0.64	
Echo	2	222	1.74 (0.29–4.06), <0.0001		
Nuclear	6	684	1.29 (0.00–4.37), 0.07	75.4, <0.0001	
CPX^b^	14	1748	2.62 (1.37–4.18), <0.0001	60.7, <0.0001	
6MWT^c^	2	9844	3.88 (3.50–4.27), <0.0001		
Time period					0.47
1981–1989	3	288	5.82 (0.09–17.1), 0.03	67.0, <0.0001	
1990–1999	14	3,006	2.65 (1.14–4.63), <0.0001	79.0, <0.0001	
2000–2009	13	2,284	1.47 (0.27–3.32), <0.0001	83.2, <0.0001	
2010–2015	10	11,308	1.60 (0.57–3.05), <0.0001	77.4, <0.0001	
Country					0.73
US & Canada	18	12,997	1.29 (0.40–2.53), <0.0001	76.1, <0.0001	
UK	9	1,313	1.94 (0.97–3.18), <0.0001	36.8, 0.12	
Other	13	2,576	3.42 (1.40–6.16), <0.0001	85.2, <0.0001	

^a^ ETT: exercise tolerance test;

^b^ CPX: cardiopulmonary exercise test;

^c^ 6MWT:6-minute walk test;

* p-interaction was calculated without including results from echo and 6MWT due to insufficient number of studies.

The assessment of biases using a sensitivity analysis of stratifying by eight sections in selection, comparability, and outcomes are presented in Table 3. While the study results are heterogeneous, there were no differences by methodological variables using meta-regression except for demonstration that outcome of interest was not present at the start of the study (p-interaction = 0.048). However, there were only three studies in the no category.

**Table 3:** Sensitivity analysis of 30-day mortality among non-cardiac surgery patients who received stress test using procedures specific to binomial data and exact methods by quality characteristics of the study, N = 40.

	# of studies	# of patients	ES (95% CI), p	I^2^, p-value	P-inter
Representativeness of the exposed cohort					0.039
Somewhat	2	181	9.45 (4.61–15.7), <0.0001	-	
Truly	38	16,705	1.80 (1.10–2.63), <0.0001	77.2, <0.0001	
Ascertainment of exposure					-
From secure record	40	16,886	1.98 (1.25–2.85), <0.0001	77.4, <0.0001	
Other	0				
Demonstration that outcome of interest was not present at start of study					0.048
Yes	37	16,527	1.76 (1.05–2.61), <0.0001	77.8, <0.0001	
No	3	359	7.48 (1.73–16.1), <0.0001	-	
Comparability of cohorts on the basis of the design or analysis (n = 8)					0.73
Yes	2	869	2.94 (1.42–4.89), <0.0001	-	
No	6	2,578	1.68 (0.25–3.98), <0.0001	72.8, <0.0001	
Comparability of cohorts on other factors (n = 8)					0.72
Yes	1	770	3.89 (2.19–6.33), <0.0001	68.8, <0.0001	
No	7	2,677	1.40 (0.17–3.40), 0.01	-	
Assessment of outcome					0.80
Independent blind	7	1,813	2.41 (0.36–5.81), <0.0001	87.9, <0.0001	
Records/ self-report	33	15,073	1.88 (1.12–2.78), <0.0001	73.4, <0.0001	
Was follow-up long enough for outcomes to occur					0.12
Yes	34	16,380	1.77 (1.03–2.66), <0.0001	, <0.0001	
No	6	506	4.10 (1.41–7.83), <0.0001	, <0.0001	
Adequacy of follow up of cohorts					0.39
Complete follow up	33	6,490	2.30 (1.37–2.85), <0.0001	85.2, 0.07	
Incomplete follow up	7	10,396	0.91 (0.00–3.11), <0.0001	73.2, <0.0001	

Selection of the non-exposed cohort was dropped from the analysis since this analysis is for single cohort of those who received stress test.

The contour plot demonstrates the scatter of effect estimates with log of 30-day mortality rate on the x-axis and corresponding standard error on the y-axis, indicating potential publication bias and small study effects (S20 Fig). An asymmetric funnel plot suggests the presence of small study effects due to methodological problems resulting in publication bias may have resulted in an overestimation of effects. The scatter of effect estimates from meta-regression models with standard error as an explanatory variable indicated asymmetry and suggests missing studies in the white area of non-significance on one side. The regression test for asymmetry was negative (p = 0.17).

## Discussion

This study is the largest systematic review and meta-analysis of pre-operative stress testing prior to non-cardiac surgery. Overall, we observed a general lack of quality of studies available to estimate pooled risk of 30-day mortality. We observed three main findings. First, the analysis of 40 studies out of which 36 without a comparator group, indicative of low methodological quality, observed that the 30-day mortality rate to be 1.98 with high heterogeneity between studies. Second, the 30-day mortality risk associated with pre-operative stress test versus no pre-operative test was inconclusive. Third, among the 1,807 studies considered for this analysis, 485 (26.8%) were excluded because they did not assess hard outcomes such as mortality, incidence of MI or incidence of heart failure. Among the 79 studies selected, only 40 (50.6%) presented 30-day mortality.

Of the 79 studies included in our analysis, there were only six studies with a comparison group of which three randomized controlled trials that met our inclusion criteria. We included two studies (THE DECREASE II and DECREASE V trials)[73, 74] for which significant doubt was raised regarding the validity of their findings, owing to multiple issues as outlined in the 2012 Erasmus MC Report that investigated these studies.[99] However, we included these studies in our analysis, since these articles were not retracted. The remainder were group cohort studies, often retrospective in nature, and did not include a comparison group of those patients who had undergone stress testing versus those who did not. Among the six studies comparing stress testing versus no stress testing, there was no significant difference in 30-day mortality between the groups, and the overall event rate was low. Notably, two of these studies were excluded from the analysis because their event rates were zero. While this conclusion suggests that stress testing has little impact on 30-day mortality in patients awaiting non-cardiac surgery, it is also clear that in the current body of literature, there are very few studies designed to adequately answer this question. Importantly, it is difficult to discern from the available studies if the results of pre-operative stress testing influenced down-stream decision making that may have led to the cancellation or postponement of surgery, or to other cardiovascular interventions that were implemented with the aim of reducing peri-operative risk. This appears to be the case in 17 studies that reported coronary revascularization prior to surgery (Table 1).

The majority of studies (21/40) included a population of patients awaiting vascular surgery. There was no significant difference in 30-day post-operative mortality among vascular surgery patients compared with other types of surgeries. However, the 30-day mortality rate tended to be higher (p = 0.08) among those who underwent lung surgeries (3.34%) compared to other types of surgeries (1.65%).

Since surgical techniques, anesthesia and peri-operative management have evolved over time, we evaluated studies relative to the time periods of patient enrollment. Three studies (288 patients) enrolled patients prior to 1990, 14 studies in the 1990s (3,006 patients) and 13 studies in the 2000s (2,284 patients). The majority of patients (11,308) included in this analysis were enrolled after 2010. These latter ten studies reflect a patient population exposed to the most contemporary techniques, with a 30-day post-operative mortality rate of 1.68.

While extracting data from these studies, we noted many incomplete descriptions of stress testing methodology. A large proportion of studies did not specify the exact type of stress test that was performed or included multiple types of stress tests into a single analysis. When nuclear or echo imaging stress tests were performed, exercise and pharmacological tests were most often reported as a single type of imaging stress test. Moreover, there was significant variability in how an abnormal stress test was defined, or it was often not defined at all. Hence, it is not surprising that the heterogeneity among these studies was high, thus limiting our ability to precisely analyze whether the type of stress testing used or abnormal results had any effect on patient outcomes. The duration of follow-up reported in the studies was not specified in many of the studies. Most of the studies did not have independent blinded or central adjudication of outcomes, but rather used record linkages that and may have been subject to reporting error.

Importantly, there was no ischemia-specific extractable aggregate data available in any of the 79 studies. Therefore, we were unable to perform stratified analysis by evidence of ischemia. This was similarly the case with peak exercise work rate or other test-specific measured variables. Notably, 17 studies included patients (251 in the sample) that underwent stress testing and subsequent revascularization prior to surgery leading to an important verification or workup bias, further limiting conclusions that could be derived from the reported results. Seven of the studies did not report whether revascularization had occurred prior to surgery.

Since the largest proportion of studies in our analysis included those in which cardiopulmonary testing (CPX) was performed, these deserve special comment. Only 14 out of 27 studies reported 30-day post-operative mortality which averaged 2.6%. Qualitatively, it appears that a higher exercise capacity, as measured by peak oxygen uptake (VO2), was associated with lower mortality and fewer short-term (<30 days) post-operative complications. Many of the studies provided evidence supporting this association; however, there was significant variation regarding the variables that were used to segregate patients into different post-operative risk tiers (e.g. ventilatory or anaerobic threshold, peak VO2, percentage of predicted maximal VO2 achieved), as well as the cutoff limits for each of these variables. As such, there was no specific variable or cutoff threshold that was consistent.

It is difficult to evaluate publication bias in this meta-analysis given the small number of trials. Based on the funnel plot of these six included studies, it is possible that the asymmetry may be due to reporting bias; however, the results may be more likely be attributable to chance given the small number of included trials, as well as some component of heterogeneity, as mentioned above.

Despite four decades of research since the inception of the multifactorial index of cardiac risk in non-cardiac surgical procedures and medical advances in nuclear stress testing, there are very limited quality data in the literature.[100] In fact, there have been only six meta-analyses examining pre-operative pharmacologic stress testing prior to non-cardiac surgery with the most recent analysis in 2006.[101–106] Due to the rapid advances in nuclear cardiology over the past few decades, three of the studies are outdated as they included planar imaging and SPECT without CT.[101–103] One meta-analysis exclusively evaluated end-stage renal disease patients undergoing kidney and/or pancreatic transplants and found that positive myocardial perfusion studies were associated with increased risk of myocardial infarction and cardiac death.[104] Two of the more recent meta-analyses have revealed that moderate to large perfusion defects portend a worse peri-operative morbidity.[105, 106] A meta-analysis evaluating thallium imaging and stress echocardiography in patients undergoing elective non-cardiac surgery[106] found a moderate to large defect and was predictive of 30-day myocardial infarcts and death (7.5% and 8.1% for stress echocardiograms and stress scintigraphy).[106]

Based on the 30-day mortality rate of 1.98% (95% CI 1.25% to 2.85%) reported in our analysis, for demonstrating stress test to be effective in reducing the mortality rate by 50%, the expected 30-day mortality rate in the non-stress test group would be between 2.97% (1.88% to 4.28%). The corresponding rate ratio will be 0.67. If the expected reduction in mortality rate was 80%, then the expected 30-day mortality rate in the non-stress group ranges between 3.56% (95% CI 2.25% to 5.13%). The corresponding rate ratio will be 0.55. For a superiority trial, with two-sided type 1 error of 0.05, power of 90%, randomized 1:1, comparing 1.98% to 2.97% stress test versus no stress test, the sample size for each group was 5,173 (total 10,346). For a superiority trial, with two-sided type 1 error of 0.05, power of 90%, randomized 1:1, comparing 1.98% to 3.56% stress test versus no stress test, the sample size for each group was 2,265 (total 4530). Importantly, the results of the stress test would need to be blinded by the study investigators in order to avoid the work up bias that is evident in several studies included in our systematic review. Such a study would be very difficult to conduct without the exclusion of patients who demonstrated stress test results that were predictive of a high short-term adverse event rate even without the performance of the planned surgery.

## Conclusion

Due to a large heterogeneity among the studies, our meta-analysis justifies the current American[2] and European guidelines[3] and as of 2018 does not support routine or indiscriminate pre-operative stress testing prior to non-cardiac surgery. Overall, the studies lacked methodological rigor that precludes our ability to draw any conclusions regarding whether or not pre-operative stress testing offers any valuable information to predict 30-day mortality following non-cardiac surgery. These studies are inadequate to assess whether judicious, clinically-driven pre-operative stress testing of any type among patients with specific symptoms or signs that suggest high-risk coronary artery disease, or among those undergoing a particular type of non-cardiac surgery, affect decisions, treatments and interventions that might mitigate post -operative mortality.

## Supporting information

S1 Fig Contour funnel plot to highlight the effect estimate and standard error of the studies included in this meta-analysis with a comparison of stress test versus no stress test, N = 6 studies. A larger study will have smaller standard error, indicative of better statistical precision and vice versa. Studies with large sample size are scattered on the top of the triangle, indicative of minimal bias. Studies with small sample size are on the base of the triangle. The contours of the funnel plot indicate the measures of significance; p<1%, 1%<<p5%, 5%<p<10% are in shades of grey and the white area indicate p>10%. Eggers test for absence of small study effects was not significant, p = 0.92, indicating that the effects seen in small studies vary from those estimated in larger studies and may be due to the instability of the effect sizes in small sizes or reporting bias.
(PDF)

S2 Fig Bias within eligible studies in a comparison of stress test versus no stress test among non-cardiac surgery patients, N = 6 studies. For each study, the presence (+) and absence (-) of a characteristic are recorded. If the characteristic was not clear in the trial, then it was marked as uncertain (?).
(PDF)

S3 Fig Meta-analysis of 30-day mortality among non-cardiac surgery patients who received stress test using standard meta-analysis method, N = 40. (PDF)

S4 Fig Meta-analysis of 30-day mortality among non-cardiac surgery patients who received stress test using procedures specific to binomial data with continuity correction, N = 40. (PDF)

S5 Fig Meta-analysis of 30-day mortality among non-cardiac surgery patients who received stress test using procedures specific to binomial data and exact methods, N = 40. (PDF)

S6 Fig Cumulative meta-analysis of 30-day mortality among non-cardiac surgery patients who received stress test using procedures by time period of enrollment, N = 40. ES is effect size- here it is %. The pooled estimate is calculated after Freeman-Tukey Double Arcsine Transformation (Freeman, M. F., and Tukey, J. W. 1950) to stabilize the variances.
(PDF)

S7 Fig Meta-analysis of 30-day mortality among non-cardiac surgery patients who received stress test using procedures by time period of publication, N = 40. Using Fig 6 specs by time period of publication. ES is effect size- here it is %. The pooled estimate is calculated after Freeman-Tukey Double Arcsine Transformation (Freeman, M. F., and Tukey, J. W. 1950) to stabilize the variances.
(PDF)

S8 Fig Meta-analysis of 30-day mortality among non-cardiac surgery patients who received stress test using procedures by study size, N = 40. Using Fig 6 specifications by time period of publication. ES is effect size- here it is %. The pooled estimate is calculated after Freeman-Tukey Double Arcsine Transformation (Freeman, M. F., and Tukey, J. W. 1950) to stabilize the variances.
(PDF)

S9 Fig Meta-analysis of 30-day mortality among non-cardiac surgery patients who received stress test using procedures by type of surgery- vascular. (PDF)

S10 Fig Meta-analysis of 30-day mortality among non-cardiac surgery patients who received stress test using procedures by type of surgery- lung. (PDF)

S11 Fig Meta-analysis of 30-day mortality among non-cardiac surgery patients who received stress test using procedures by type of surgery- thoracic, not lung. (PDF)

S12 Fig Meta-analysis of 30-day mortality among non-cardiac surgery patients who received stress test using procedures by type of surgery- abdominal. (PDF)

S13 Fig Meta-analysis of 30-day mortality among non-cardiac surgery patients who received stress test using procedures by type of surgery- gynecological. (PDF)

S14 Fig Meta-analysis of 30-day mortality among non-cardiac surgery patients who received stress test using procedures by type of surgery- urological. (PDF)

S15 Fig Meta-analysis of 30-day mortality among non-cardiac surgery patients who received stress test using procedures by type of surgery- renal transplant. (PDF)

S16 Fig Meta-analysis of 30-day mortality among non-cardiac surgery patients who received stress test using procedures by type of surgery- liver transplant. (PDF)

S17 Fig Meta-analysis of 30-day mortality among non-cardiac surgery patients who received stress test using procedures by type of surgery- orthopedic. (PDF)

S18 Fig Meta-analysis of 30-day mortality among non-cardiac surgery patients who received stress test using procedures by type of surgery- gastric bypass. (PDF)

S19 Fig Meta-analysis of 30-day mortality among non-cardiac surgery patients who received stress test using procedures by type of surgery- other. (PDF)

S20 Fig Contour funnel plot to highlight the effect estimate and standard error of 30-day mortality related to stress test in studies included in this meta-analysis, N = 40 studies. Eggers test = 0.17.
(PDF)

S1 Appendix Search criteria. (PDF)

S2 Appendix Phase 1 screening or Abstract screening inclusion and exclusion criteria. (PDF)

S3 Appendix Results of phase 1 screening or abstract screening. (PDF)

S4 Appendix Phase 2 screening or full-text screening inclusion and exclusion criteria. (PDF)

S5 Appendix Newcastle Ottawa Quality Assessment Scale. (PDF)

S1 Checklist PRISMA 2009 checklist. (DOC)
