# Supplementary material for: Pre-operative stress testing in the evaluation of patients undergoing non-cardiac surgery: A systematic review and meta-analysis
Source: PLoS One. 2019 Jul 11;14(7):e0219145. doi: 10.1371/journal.pone.0219145 (PMC6622497; doi:10.1371/journal.pone.0219145)
Supplement: S1 Fig — A larger study will have smaller standard error, indicative of better statistical precision and vice versa. Studies with large sample size are scattered on the top of the triangle, indicative of minimal bias. Studies with small sample size are on the base of the triangle. The contours of the funnel plot indicate the measures of significance; p<1%, 1%<<p5%, 5%<p<10% are in shades of grey and the white area indicate p>10%. Eggers test for absence of small study effects was not significant, p = 0.92, indicating that the effects seen in small studies vary from those estimated in larger studies and may be due to the instability of the effect sizes in small sizes or reporting bias. (PDF) [file pone.0219145.s001.pdf]

Pre-operative stress testing in the evaluation of patients undergoing non-cardiac surgery: A systematic review and meta-analysis

S1 Fig: Contour funnel plot to highlight the effect estimate and standard error of the studies included in this meta-analysis with a comparison of stress test versus no stress test, N=6 studies

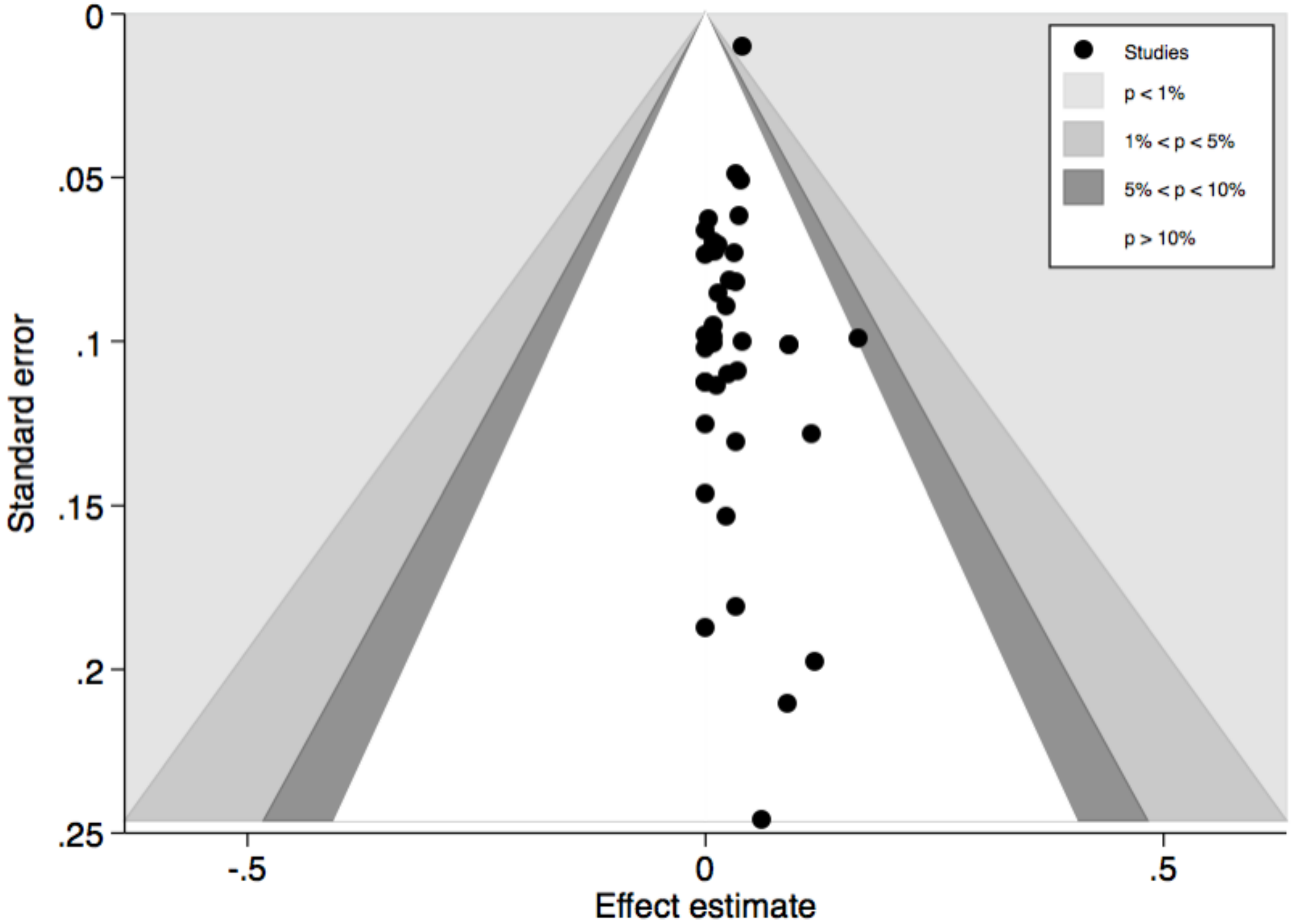

A larger study will have smaller standard error, indicative of better statistical precision and vice versa. Studies with large sample size are scattered on the top of the triangle, indicative of minimal bias. Studies with small sample size are on the base of the triangle. The contours of the funnel plot indicate the measures of significance; p<1%, 1%<<p5%, 5%<p<10% are in shades of grey and the white area indicate p>10%. Eggers test for absence of small study effects was not significant, p=0.92, indicating that the effects seen in small studies vary from those estimated in larger studies and may be due to the instability of the effect sizes in small sizes or reporting bias.
