# Supplementary material for: Pre-operative stress testing in the evaluation of patients undergoing non-cardiac surgery: A systematic review and meta-analysis
Source: PLoS One. 2019 Jul 11;14(7):e0219145. doi: 10.1371/journal.pone.0219145 (PMC6622497; doi:10.1371/journal.pone.0219145)
Supplement: S2 Fig — For each study, the presence (+) and absence (-) of a characteristic are recorded. If the characteristic was not clear in the trial, then it was marked as uncertain (?). (PDF) [file pone.0219145.s002.pdf]

Pre-operative stress testing in the evaluation of patients undergoing non-cardiac surgery: A systematic review and meta-analysis

S2 Fig: Bias within eligible studies in a comparison of stress test versus no stress test among non-cardiac surgery patients, N=6 studies

	Intent-to-treat	Concealment of allocation	Outcomes adjudicated independently		Representativeness of intervention	Selection of intervention	Ascertainment of intervention	Outcome not present at start	Comparability of cohorts, adjusted for main factors	Comparability of cohorts, adjusted for other factors	Assessment of outcome	Mean follow up duration >=30days	Adequate follow up duration
Marjanski					+	+	+	+	-	-	+	+	+
Afolabi					+	?	+	+	?	?	+	+	-
Falcone	+	+	+										
Fleisher					+	+	+	+	-	-	+	+	+
Erikson					+	+	+	-	-	-	+	+	+
Poldermans	+	+	+										

For each study, the presence (+) and absence (-) of a characteristic are recorded. If the characteristic was not clear in the trial, then it was marked as uncertain (?)
