# Supplementary material for: Pre-operative stress testing in the evaluation of patients undergoing non-cardiac surgery: A systematic review and meta-analysis
Source: PLoS One. 2019 Jul 11;14(7):e0219145. doi: 10.1371/journal.pone.0219145 (PMC6622497; doi:10.1371/journal.pone.0219145)

S3 Fig: Meta-analysis of 30-day mortality among non-cardiac surgery patients who received stress test using standard meta-analysis method, N=40

Studies	year	StressTest	ES (95% CI)	% Weight
Smith, T.P.	1984	2/22	0.09 (-0.03, 0.21)	0.47
Carliner, N.	1985	3/200	0.01 (-0.00, 0.03)	4.64
Lette J.	1989	7/60	0.12 (0.04, 0.20)	0.93
Joyce, W	1990	1/77	0.01 (-0.01, 0.04)	3.79
Holley	1991	1/189	0.01 (-0.01, 0.02)	5.22
Holden	1992	1/16	0.06 (-0.06, 0.18)	0.48
Davila-Roman, V.G.	1993	1/98	0.01 (-0.01, 0.03)	4.34
Epstein, S.K.	1993	1/42	0.02 (-0.02, 0.07)	2.15
Kaaja, R	1993	2/58	0.03 (-0.01, 0.08)	2.10
Bollinger, C.T	1995	3/25	0.12 (-0.01, 0.25)	0.42
Erickson, C.E.	1996	5/147	0.03 (0.00, 0.06)	3.41
Larsen, K.R.	1997	9/97	0.09 (0.04, 0.15)	1.59
Richter Larsen, K.	1997	9/97	0.09 (0.04, 0.15)	1.59
Nugent	1998	1/30	0.03 (-0.03, 0.10)	1.36
Fleisher, L.A.	1999	10/261	0.04 (0.02, 0.06)	3.99
Gauss, A.	1999	2/204	0.01 (-0.00, 0.02)	4.96
Mondillo, S	2002	2/188	0.01 (-0.00, 0.03)	4.85
Villani, F	2003	4/150	0.03 (0.00, 0.05)	3.75
Win, T	2005	4/99	0.04 (0.00, 0.08)	2.63
McMcCullough, P.A.	2006	1/109	0.01 (-0.01, 0.03)	4.54
Poldermans, D.	2006	15/386	0.04 (0.02, 0.06)	4.40
Poldermans, D.	2007	17/101	0.17 (0.10, 0.24)	1.11
Shouten, O.	2008	3/124	0.02 (-0.00, 0.05)	3.62
Thompson, A.R.	2011	1/102	0.01 (-0.01, 0.03)	4.42
Hartley	2012	14/415	0.03 (0.02, 0.05)	4.59
Prentis, J.M.	2012	6/185	0.03 (0.01, 0.06)	3.77
James,S	2013	3/83	0.04 (-0.00, 0.08)	2.53
Prentis, J.M.	2013	2/82	0.02 (-0.01, 0.06)	3.05
West, M.A.	2014	2/136	0.01 (-0.01, 0.03)	4.30
Castleberry, A.W.	2015	390/9526	0.04 (0.04, 0.04)	5.58
Marjanski T.	2015	1/253	0.00 (-0.00, 0.01)	5.40
Reifsnyder, T.	1992	0/103	(Excluded)	0.00
Falcone, R.A.	2003	0/46	(Excluded)	0.00
Golzar, JA	2004	0/63	(Excluded)	0.00
Park, K.W.	2005	0/28	(Excluded)	0.00
Forshaw, M.J.	2008	0/78	(Excluded)	0.00
Jaroszewski, D	2008	0/184	(Excluded)	0.00
Afolabi, B.A.	2009	0/78	(Excluded)	0.00
Aalten, J.	2011	0/227	(Excluded)	0.00
West, M.A.	2014	0/95	(Excluded)	0.00
Overall (I-squared = 81.2%, p = 0.000)			0.03 (0.02, 0.04)	100.00

NOTE: Weights are from random effects analysis

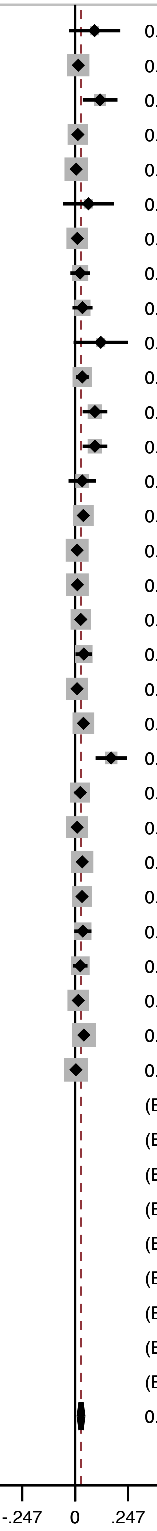

Supplement: S3 Fig — (PDF) [file pone.0219145.s003.pdf]
