# Supplementary material for: Pre-operative stress testing in the evaluation of patients undergoing non-cardiac surgery: A systematic review and meta-analysis
Source: PLoS One. 2019 Jul 11;14(7):e0219145. doi: 10.1371/journal.pone.0219145 (PMC6622497; doi:10.1371/journal.pone.0219145)

S4 Fig: Meta-analysis of 30-day mortality among non-cardiac surgery patients who received stress test using procedures specific to binomial data with continuity correction, N=40

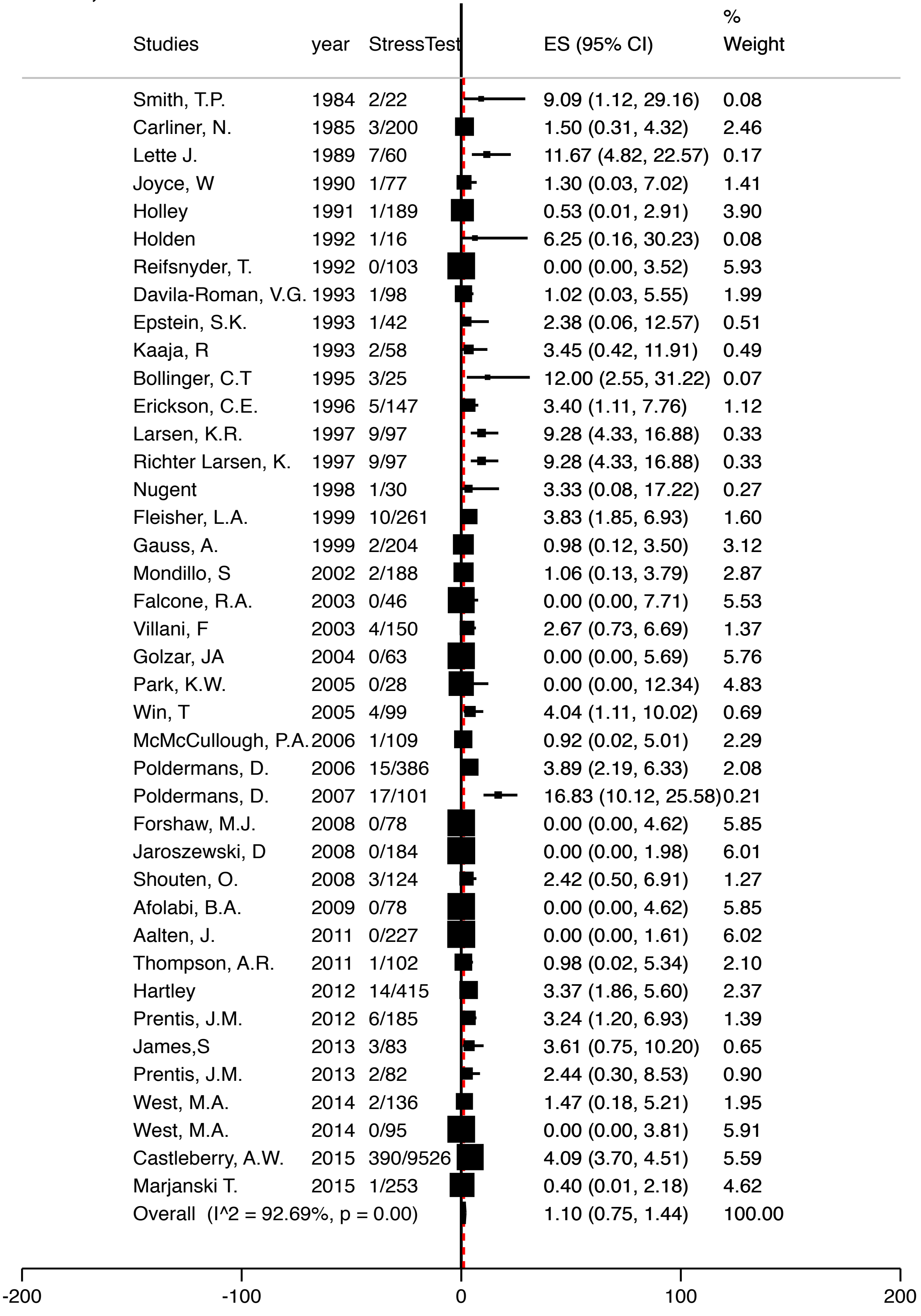

Supplement: S4 Fig — (PDF) [file pone.0219145.s004.pdf]
