# Supplementary material for: Pre-operative stress testing in the evaluation of patients undergoing non-cardiac surgery: A systematic review and meta-analysis
Source: PLoS One. 2019 Jul 11;14(7):e0219145. doi: 10.1371/journal.pone.0219145 (PMC6622497; doi:10.1371/journal.pone.0219145)
Supplement: S5 Fig — (PDF) [file pone.0219145.s005.pdf]

S5 Fig: Meta-analysis of 30-day mortality among non-cardiac surgery patients who received stress test using procedures specific to binomial data and exact methods, N=40

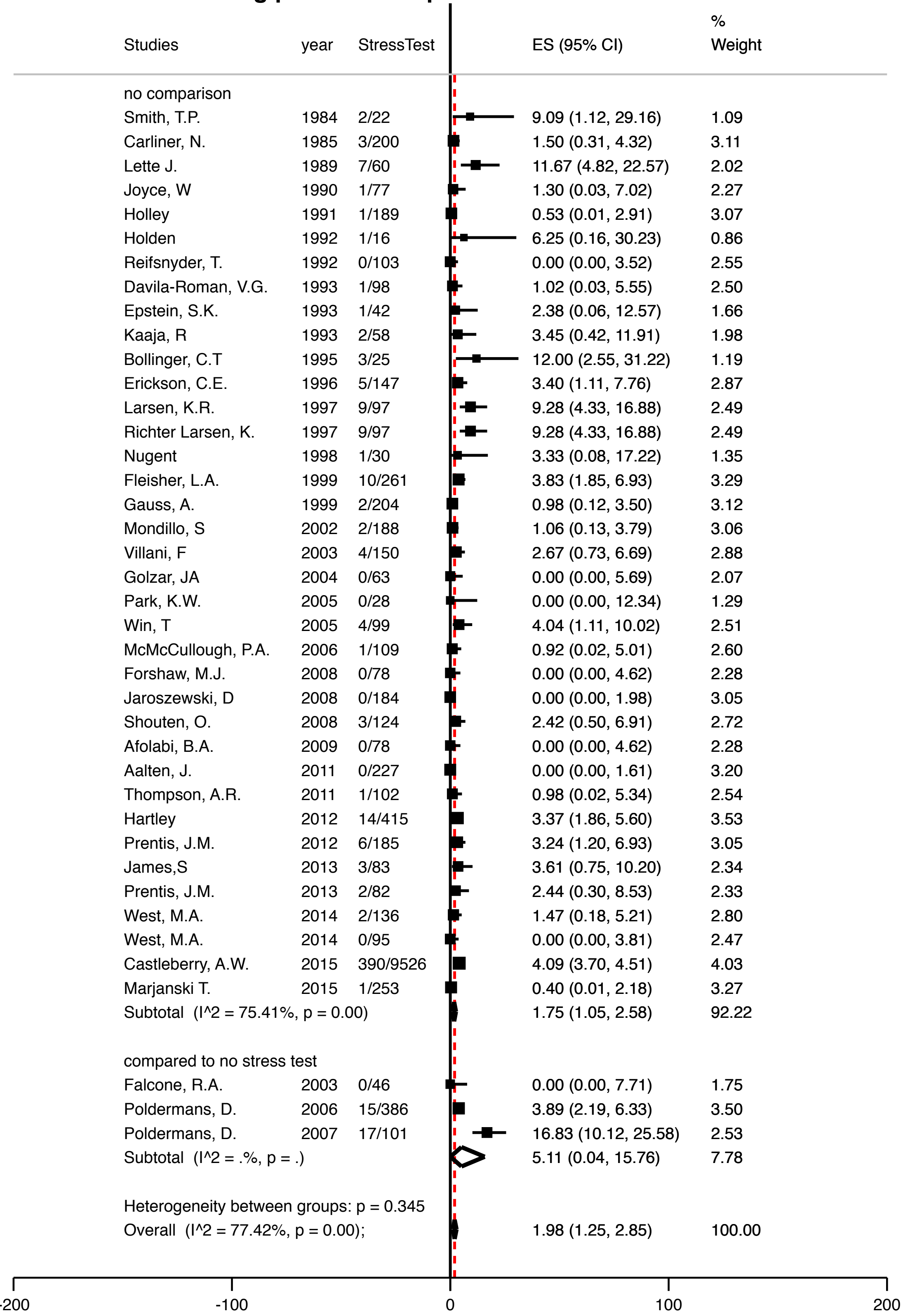

Studies	year	StressTest	ES (95% CI)	% Weight
no comparison				
Smith, T.P.	1984	2/22	9.09 (1.12, 29.16)	1.09
Carliner, N.	1985	3/200	1.50 (0.31, 4.32)	3.11
Lette J.	1989	7/60	11.67 (4.82, 22.57)	2.02
Joyce, W	1990	1/77	1.30 (0.03, 7.02)	2.27
Holley	1991	1/189	0.53 (0.01, 2.91)	3.07
Holden	1992	1/16	6.25 (0.16, 30.23)	0.86
Reifsnyder, T.	1992	0/103	0.00 (0.00, 3.52)	2.55
Davila-Roman, V.G.	1993	1/98	1.02 (0.03, 5.55)	2.50
Epstein, S.K.	1993	1/42	2.38 (0.06, 12.57)	1.66
Kaaja, R	1993	2/58	3.45 (0.42, 11.91)	1.98
Bollinger, C.T	1995	3/25	12.00 (2.55, 31.22)	1.19
Erickson, C.E.	1996	5/147	3.40 (1.11, 7.76)	2.87
Larsen, K.R.	1997	9/97	9.28 (4.33, 16.88)	2.49
Richter Larsen, K.	1997	9/97	9.28 (4.33, 16.88)	2.49
Nugent	1998	1/30	3.33 (0.08, 17.22)	1.35
Fleisher, L.A.	1999	10/261	3.83 (1.85, 6.93)	3.29
Gauss, A.	1999	2/204	0.98 (0.12, 3.50)	3.12
Mondillo, S	2002	2/188	1.06 (0.13, 3.79)	3.06
Villani, F	2003	4/150	2.67 (0.73, 6.69)	2.88
Golzar, JA	2004	0/63	0.00 (0.00, 5.69)	2.07
Park, K.W.	2005	0/28	0.00 (0.00, 12.34)	1.29
Win, T	2005	4/99	4.04 (1.11, 10.02)	2.51
McMcCullough, P.A.	2006	1/109	0.92 (0.02, 5.01)	2.60
Forshaw, M.J.	2008	0/78	0.00 (0.00, 4.62)	2.28
Jaroszewski, D	2008	0/184	0.00 (0.00, 1.98)	3.05
Shouten, O.	2008	3/124	2.42 (0.50, 6.91)	2.72
Afolabi, B.A.	2009	0/78	0.00 (0.00, 4.62)	2.28
Aalten, J.	2011	0/227	0.00 (0.00, 1.61)	3.20
Thompson, A.R.	2011	1/102	0.98 (0.02, 5.34)	2.54
Hartley	2012	14/415	3.37 (1.86, 5.60)	3.53
Prentis, J.M.	2012	6/185	3.24 (1.20, 6.93)	3.05
James,S	2013	3/83	3.61 (0.75, 10.20)	2.34
Prentis, J.M.	2013	2/82	2.44 (0.30, 8.53)	2.33
West, M.A.	2014	2/136	1.47 (0.18, 5.21)	2.80
West, M.A.	2014	0/95	0.00 (0.00, 3.81)	2.47
Castleberry, A.W.	2015	390/9526	4.09 (3.70, 4.51)	4.03
Marjanski T.	2015	1/253	0.40 (0.01, 2.18)	3.27
Subtotal (I^2 = 75.41%, p = 0.00)			1.75 (1.05, 2.58)	92.22
compared to no stress test				
Falcone, R.A.	2003	0/46	0.00 (0.00, 7.71)	1.75
Poldermans, D.	2006	15/386	3.89 (2.19, 6.33)	3.50
Poldermans, D.	2007	17/101	16.83 (10.12, 25.58)	2.53
Subtotal (I^2 = .%, p = .)			5.11 (0.04, 15.76)	7.78
Heterogeneity between groups: p = 0.345				
Overall (I^2 = 77.42%, p = 0.00);			1.98 (1.25, 2.85)	100.00
