# Supplementary material for: Pre-operative stress testing in the evaluation of patients undergoing non-cardiac surgery: A systematic review and meta-analysis
Source: PLoS One. 2019 Jul 11;14(7):e0219145. doi: 10.1371/journal.pone.0219145 (PMC6622497; doi:10.1371/journal.pone.0219145)
Supplement: S6 Fig — ES is effect size- here it is %. The pooled estimate is calculated after Freeman-Tukey Double Arcsine Transformation (Freeman, M. F., and Tukey, J. W. 1950) to stabilize the variances. (PDF) [file pone.0219145.s006.pdf]

S6 Fig: Cumulative meta-analysis of 30-day mortality among non-cardiac surgery patients who received stress test using procedures by time period of enrollment, N=40

Studies	year	StressTest	ES (95% CI)
Cutler, B.S.	1981	./.	-4.20 (-4.34, -4.06)
Smith, T.P.	1984	2/22	-3.31 (-5.08, -1.54)
Arous, EJ	1984	./.	-3.31 (-5.08, -1.54)
Carliner, N.	1985	3/200	-4.20 (-4.34, -4.06)
McPhail, N.	1988	./.	-3.28 (-4.35, -2.21)
Lette J.	1989	7/60	-2.92 (-4.45, -1.39)
McPhail, N.	1989	./.	-3.28 (-4.35, -2.21)
Boysen,P.G.	1990	./.	-3.31 (-5.08, -1.54)
Joyce, W	1990	1/77	-3.28 (-4.35, -2.21)
Holley	1991	1/189	-3.58 (-4.27, -2.89)
Reifsnyder, T.	1992	0/103	-3.28 (-4.35, -2.21)
Holden	1992	1/16	-3.50 (-4.16, -2.85)
Epstein, S.K.	1993	1/42	-3.17 (-4.00, -2.33)
Older P.	1993	./.	-3.17 (-4.00, -2.33)
Kaaja, R	1993	2/58	-3.20 (-3.90, -2.49)
Davila-Roman, V.G.	1993	1/98	-3.37 (-4.02, -2.73)
Seeger, J.M.	1994	./.	-3.05 (-4.05, -2.05)
Bollinger, C.T	1995	3/25	-3.05 (-4.05, -2.05)
Mocini, D	1995	./.	-3.58 (-4.27, -2.89)
Pellikka, P.A.	1996	./.	-3.50 (-4.16, -2.85)
Donovan, C.L	1996	./.	-3.40 (-4.08, -2.72)
Erickson, C.E.	1996	5/147	-3.40 (-3.89, -2.91)
Richter Larsen, K.	1997	9/97	-3.40 (-4.08, -2.72)
Van Damme H.	1997	./.	-3.40 (-4.04, -2.76)
Larsen, K.R.	1997	9/97	-3.32 (-3.95, -2.68)
Deville, C.	1997	./.	-3.41 (-4.00, -2.83)
Poldermans, D.	1997	./.	-3.40 (-3.89, -2.91)
D'Angelo, A.J.	1997	./.	-3.40 (-3.89, -2.91)
Kryzhanovski, V.A.	1997	./.	-3.40 (-3.89, -2.91)
Nugent	1998	1/30	-3.40 (-4.04, -2.76)
Won, A	1998	./.	-3.40 (-3.89, -2.91)
Gauss, A.	1999	2/204	-3.41 (-4.00, -2.83)
Fleisher, L.A.	1999	10/261	-3.40 (-3.93, -2.88)
Williams, K.	2000	./.	-3.50 (-4.16, -2.85)
Das, M.K.	2000	./.	-3.41 (-4.00, -2.83)
Lacroix, H.	2000	./.	-3.40 (-3.89, -2.91)
Farid, I.	2002	./.	-3.41 (-3.84, -2.98)
Mondillo, S	2002	2/188	-3.47 (-3.88, -3.05)
Villani, F	2003	4/150	-3.42 (-3.87, -2.97)
Falcone, R.A.	2003	0/46	-3.41 (-3.84, -2.98)
Ali, M	2004	./.	-3.41 (-3.84, -2.98)
Epstein, S.K.	2004	./.	-3.41 (-3.84, -2.98)
Golzar, JA	2004	0/63	-3.41 (-3.84, -2.98)
Park, K.W.	2005	0/28	-3.40 (-3.89, -2.91)
Win, T	2005	4/99	-3.41 (-3.84, -2.98)
Poldermans, D.	2006	15/386	-3.46 (-3.84, -3.08)
McMcCullough, P.A.	2006	1/109	-3.44 (-3.83, -3.04)
Bai, J.	2007	./.	-3.47 (-3.88, -3.05)
Poldermans, D.	2007	17/101	-3.38 (-3.78, -2.97)
Gonzalez, CA	2008	./.	-3.46 (-3.84, -3.08)
Forshaw, M.J.	2008	0/78	-3.46 (-3.84, -3.08)
Shouten, O.	2008	3/124	-3.45 (-3.82, -3.07)
Jaroszewski, D	2008	0/184	-3.45 (-3.82, -3.07)
Brunelli, A.	2009	./.	-3.45 (-3.82, -3.07)
Kasikcioglu, E.	2009	./.	-3.45 (-3.82, -3.07)
Afolabi, B.A.	2009	0/78	-3.45 (-3.82, -3.07)
Matyal R.	2010	./.	-3.47 (-3.88, -3.05)
Wijeysundera, D.N.	2010	./.	-3.38 (-3.78, -2.97)
Snowden, C.P.	2010	./.	-3.45 (-3.82, -3.07)
Thompson, A.R.	2011	1/102	-3.50 (-3.86, -3.13)
Aalten, J.	2011	0/227	-3.50 (-3.86, -3.13)
Bub, G.L	2011	./.	-3.50 (-3.84, -3.16)
Shetty, V.J.	2011	./.	-3.58 (-3.85, -3.30)
Koh, A.S.	2012	./.	-3.45 (-3.82, -3.07)
Prentis, J.M.	2012	./.	-3.50 (-3.86, -3.13)
Hartley	2012	14/415	-3.59 (-3.93, -3.25)
Prentis, J.M.	2012	6/185	-3.59 (-3.91, -3.26)
Ausania, F	2012	./.	-3.58 (-3.85, -3.30)
James,S	2013	3/83	-3.49 (-3.85, -3.14)
Prentis, J.M.	2013	2/82	-3.50 (-3.84, -3.16)
Grant, SW	2014	./.	-3.49 (-3.85, -3.14)
West, M.A.	2014	0/95	-3.49 (-3.85, -3.14)
West, M.A.	2014	2/136	-3.53 (-3.86, -3.20)
Snipelisky, D.	2014	./.	-3.58 (-3.85, -3.30)
Tolchard S.	2015	./.	-3.50 (-3.84, -3.16)
Ulyett, S.	2015	./.	-3.50 (-3.84, -3.16)
Marjanski T.	2015	1/253	-3.60 (-3.96, -3.24)
Chaikriangkrai, K.	2015	./.	-3.59 (-3.93, -3.25)
Castleberry, A.W.	2015	390/9526	-3.58 (-3.85, -3.30)

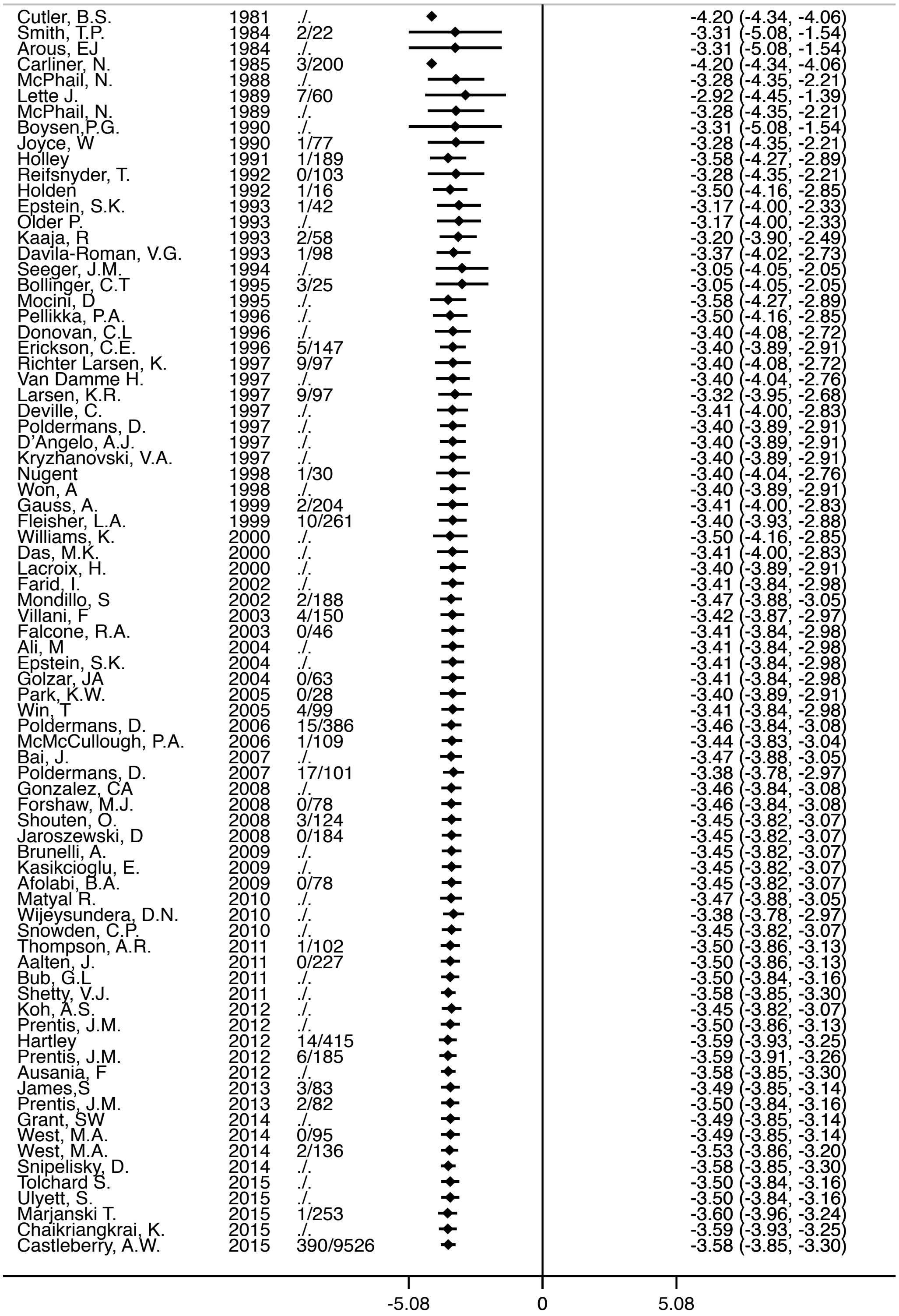

ES is effect size- here it is %. The pooled estimate is calculated after Freeman-Tukey Double Arcsine Transformation (Freeman, M. F., and Tukey, J. W. 1950) to stabilize the variances.
