# Supplementary material for: Pre-operative stress testing in the evaluation of patients undergoing non-cardiac surgery: A systematic review and meta-analysis
Source: PLoS One. 2019 Jul 11;14(7):e0219145. doi: 10.1371/journal.pone.0219145 (PMC6622497; doi:10.1371/journal.pone.0219145)
Supplement: S7 Fig — Using Fig 6 specs by time period of publication. ES is effect size- here it is %. The pooled estimate is calculated after Freeman-Tukey Double Arcsine Transformation (Freeman, M. F., and Tukey, J. W. 1950) to stabilize the variances. (PDF) [file pone.0219145.s007.pdf]

S7 Fig: Meta-analysis of 30-day mortality among non-cardiac surgery patients who received stress test using procedures by time period of publication, N=40

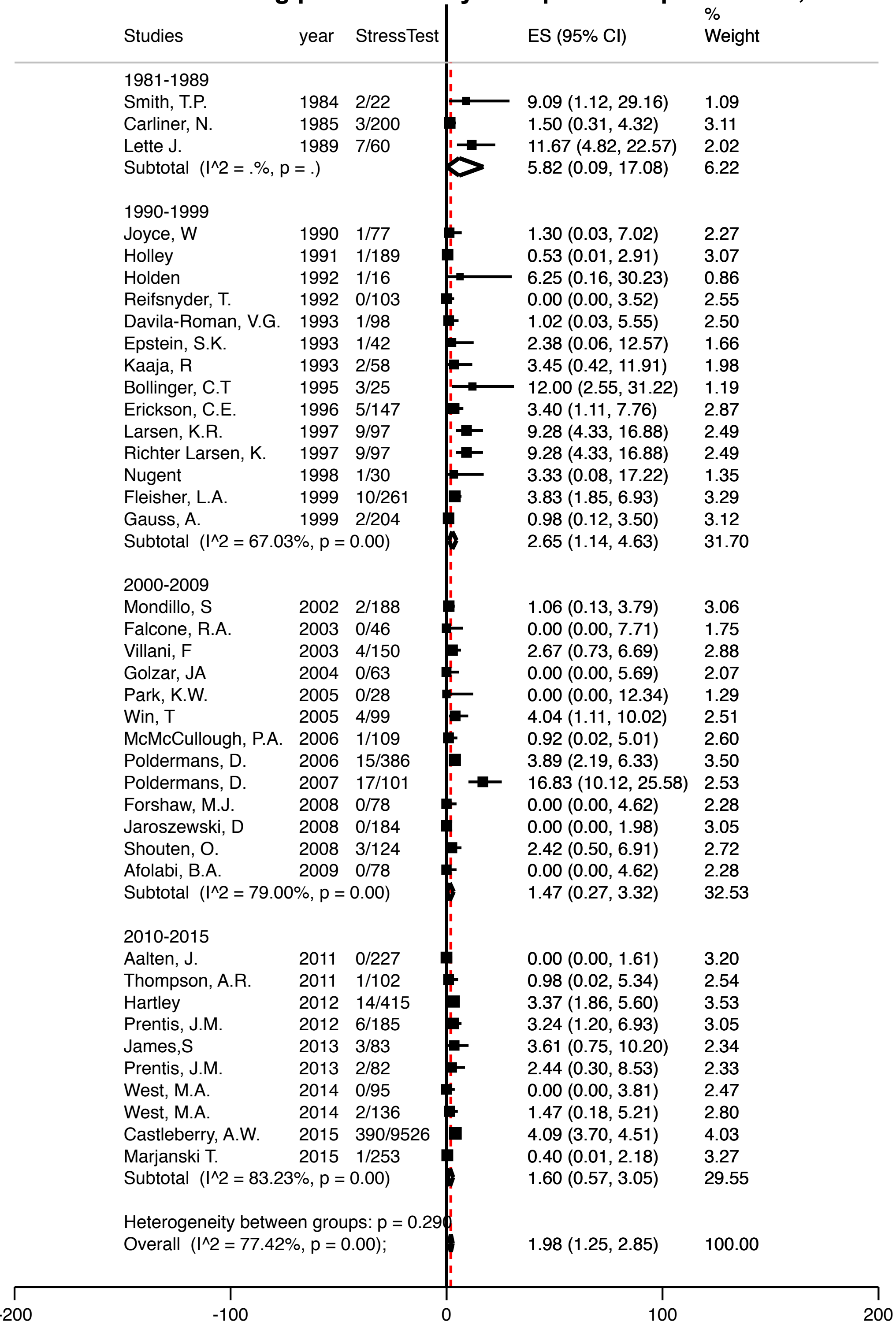

Using Figure 6 specs by time period of publication. ES is effect size- here it is %. The pooled estimate is calculated after Freeman-Tukey Double Arcsine Transformation (Freeman, M. F., and Tukey, J. W. 1950) to stabilize the variances.
