# Supplementary figures and images for: Pre-operative stress testing in the evaluation of patients undergoing non-cardiac surgery: A systematic review and meta-analysis
Source: PLoS One. 2019 Jul 11;14(7):e0219145. doi: 10.1371/journal.pone.0219145 (PMC6622497; doi:10.1371/journal.pone.0219145)

# Supplementary Figure 9: By type of surgery- vascular

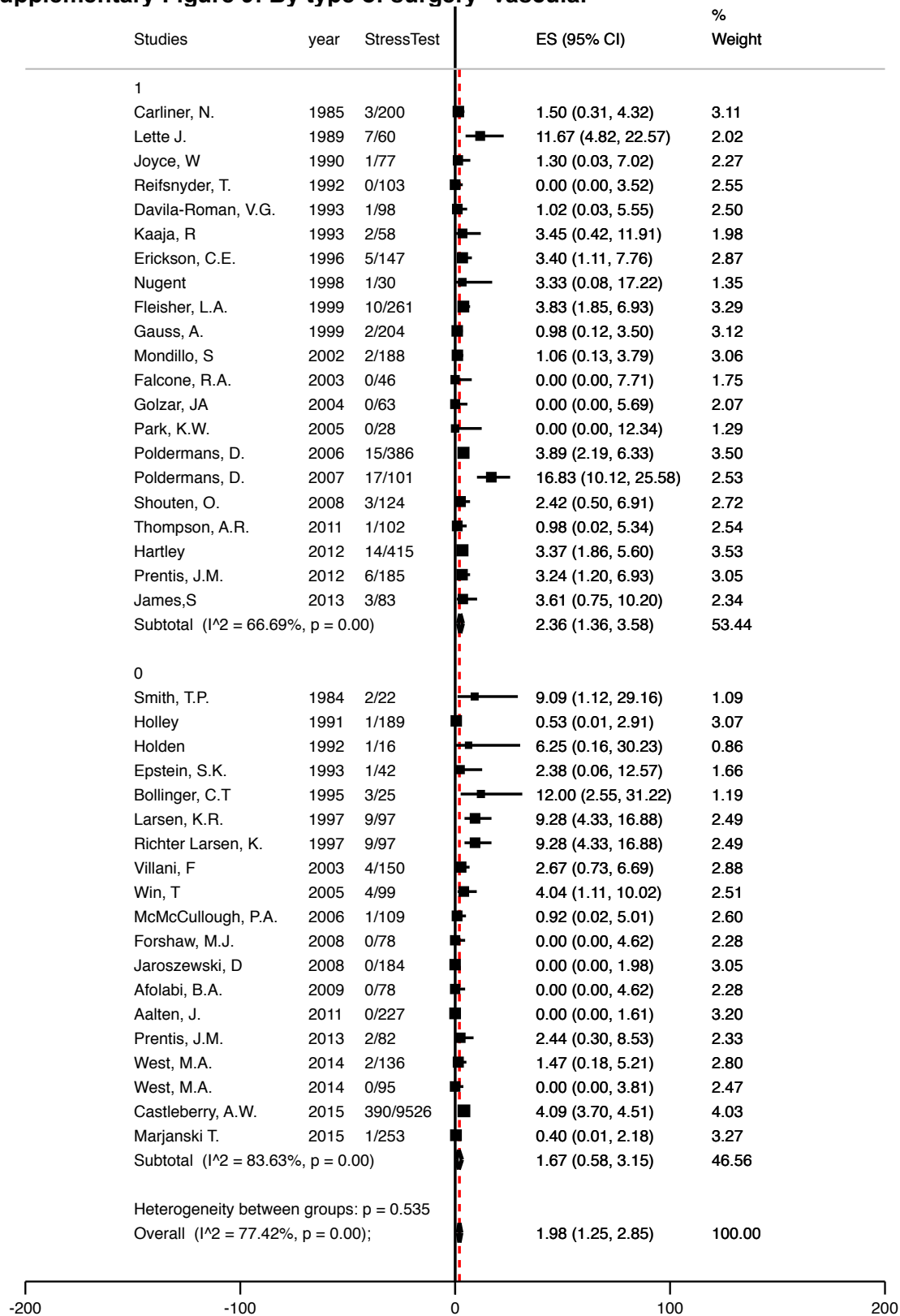

Supplement: S9 Fig — (PDF) [file pone.0219145.s009.pdf]

# Supplementary 10: By type of surgery- lung

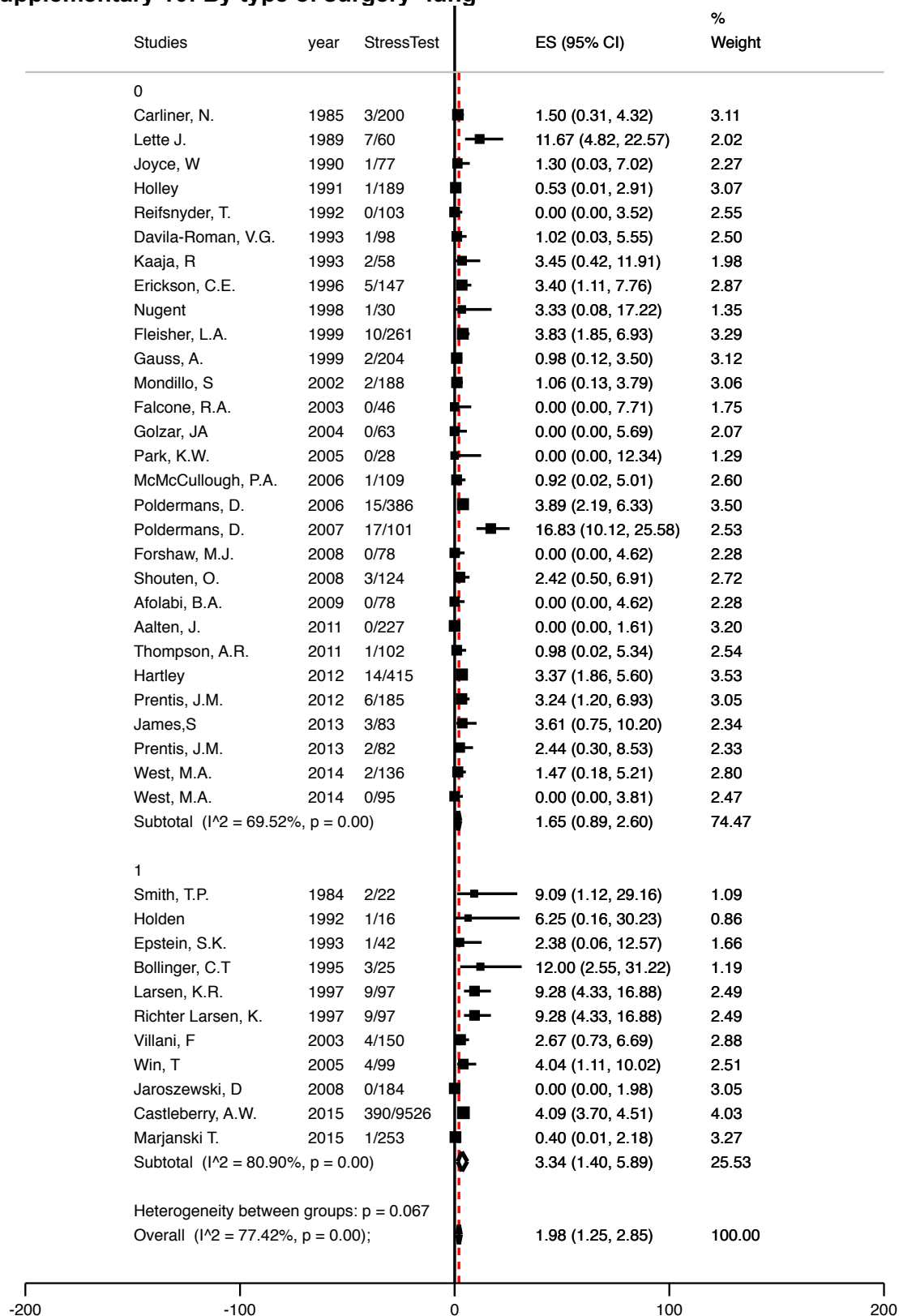

Supplement: S10 Fig — (PDF) [file pone.0219145.s010.pdf]

**Supplementary Figure 11: By type of surgery- thoracic, not lung**

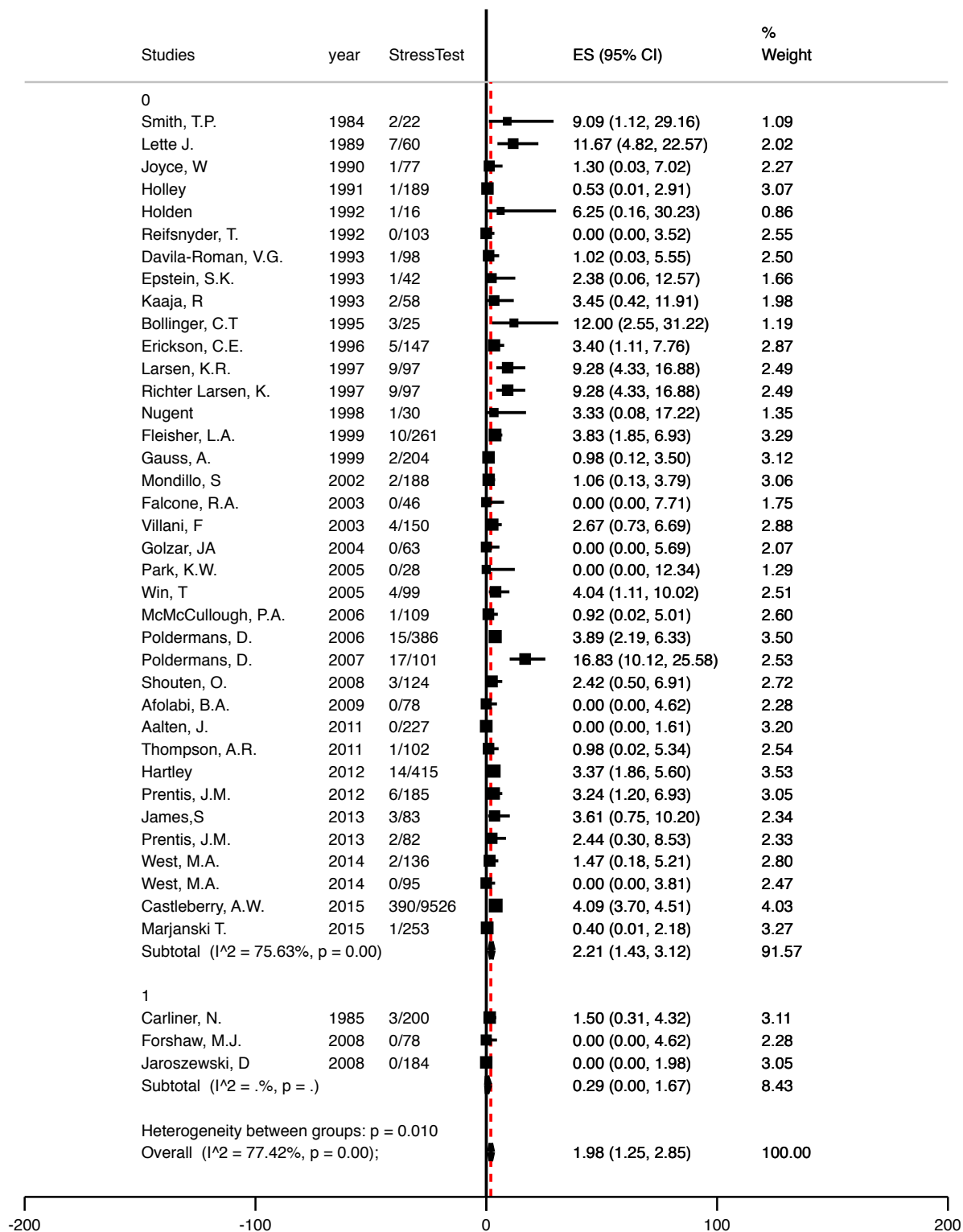

Supplement: S11 Fig — (PDF) [file pone.0219145.s011.pdf]

# Supplementary Figure 12: By type of surgery- abdominal

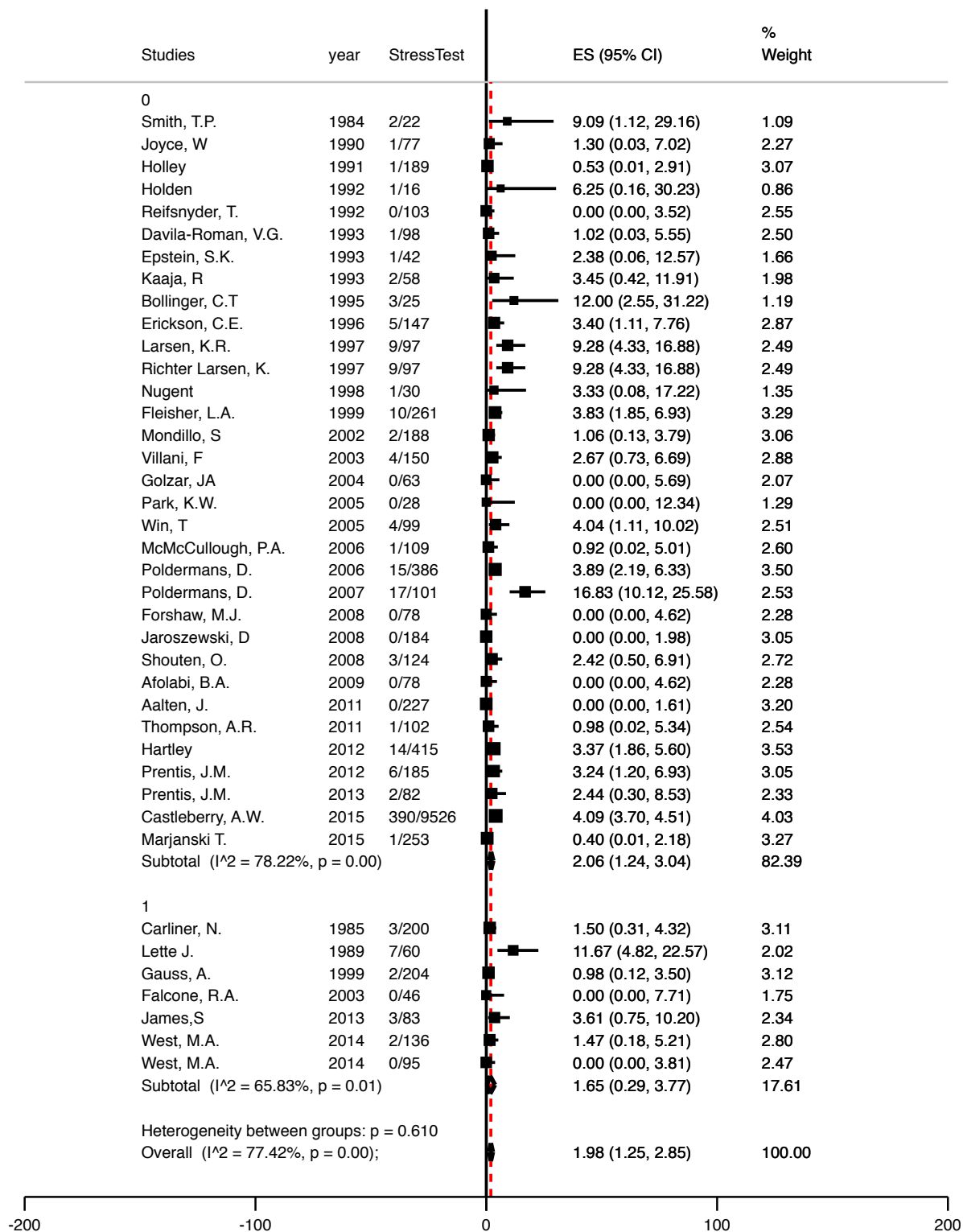

Supplement: S12 Fig — (PDF) [file pone.0219145.s012.pdf]

# Supplementary Figure 13: By type of surgery- gynecological

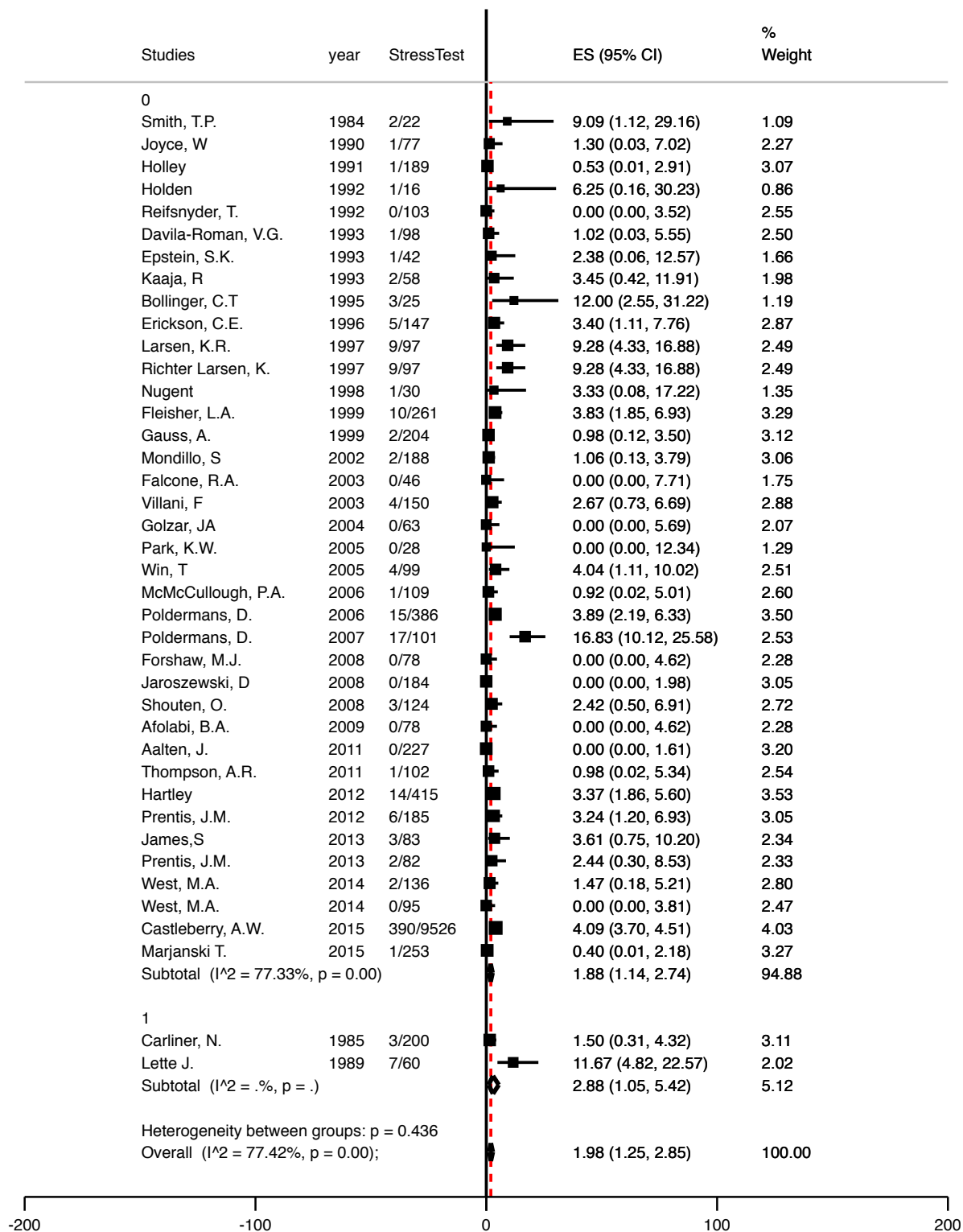

Supplement: S13 Fig — (PDF) [file pone.0219145.s013.pdf]

# Supplementary Figure 14: By type of surgery- urological

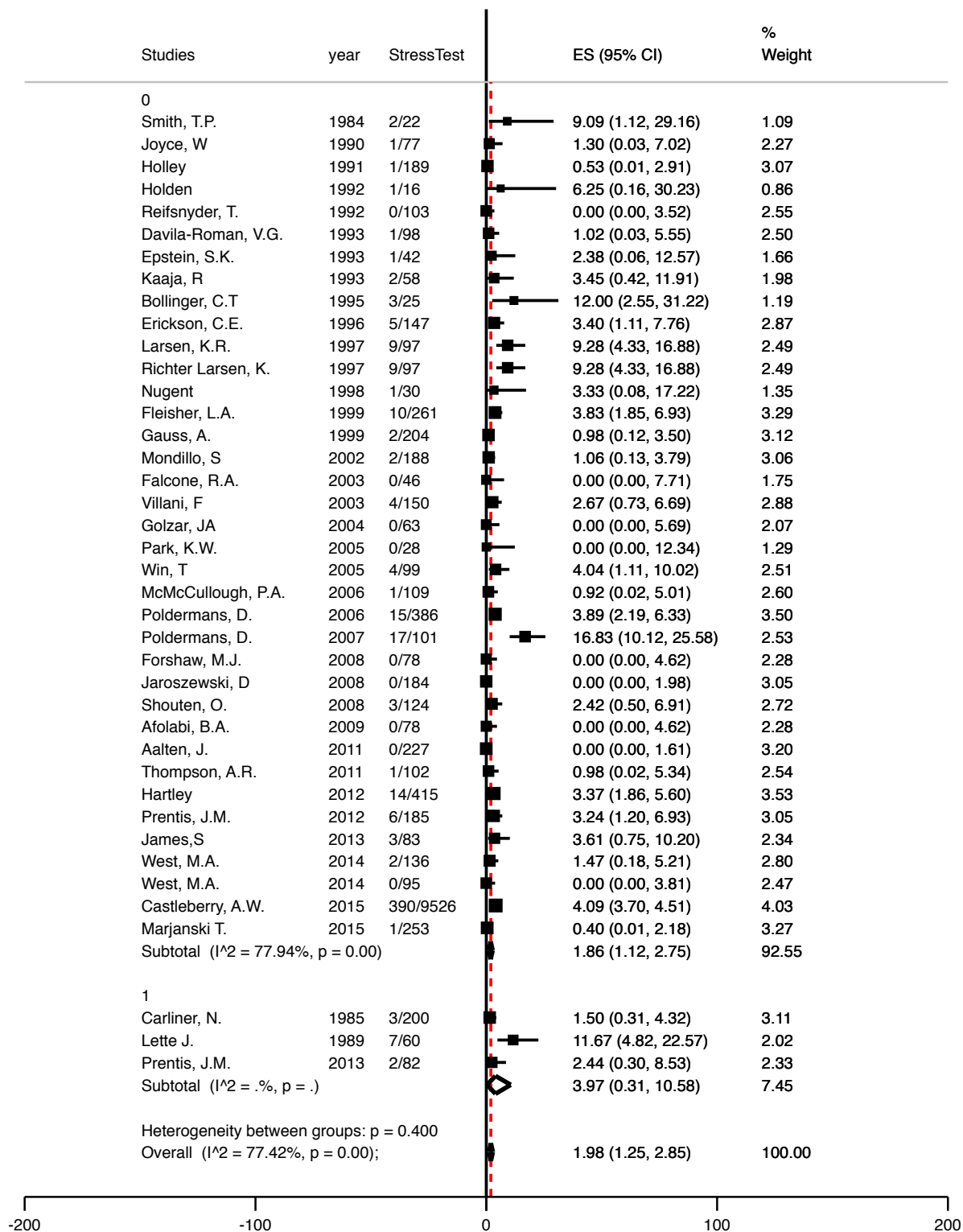

Supplement: S14 Fig — (PDF) [file pone.0219145.s014.pdf]

# Supplementary Figure 15: By type of surgery- renal transplant

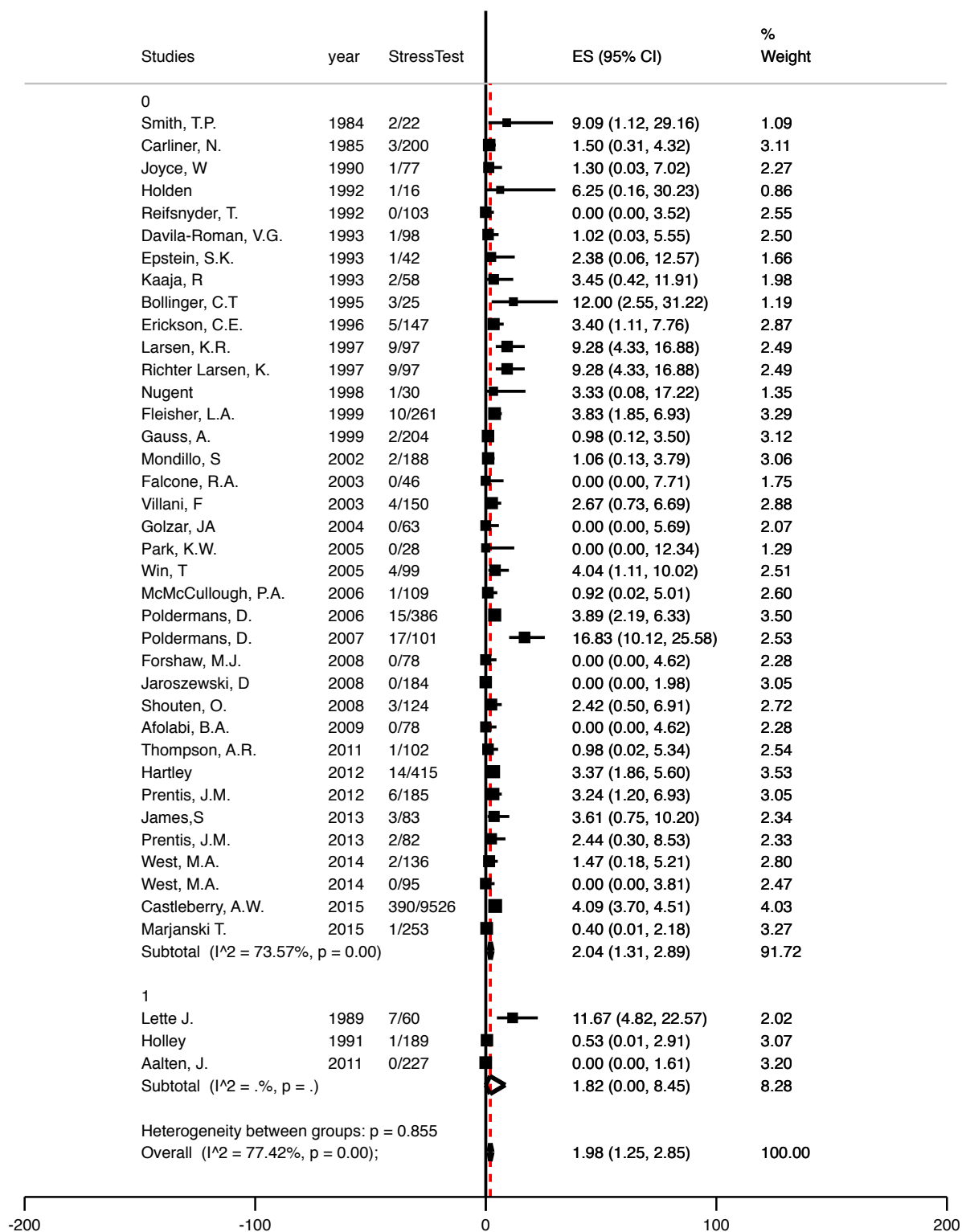

Supplement: S15 Fig — (PDF) [file pone.0219145.s015.pdf]

**Supplementary Figure 16: By type of surgery- liver transplant**

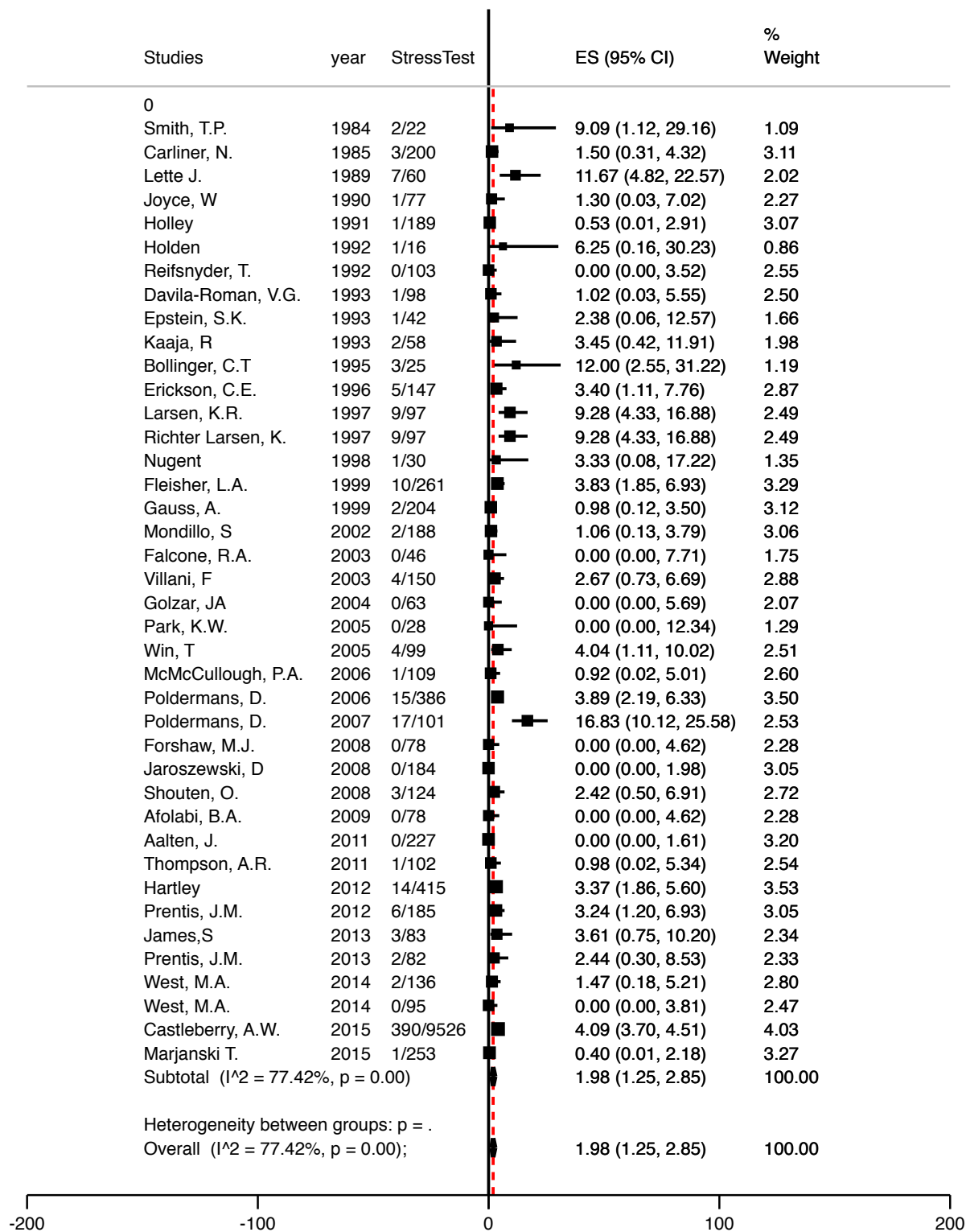

Supplement: S16 Fig — (PDF) [file pone.0219145.s016.pdf]

# Supplementary Figure 17: By type of surgery- orthopedic

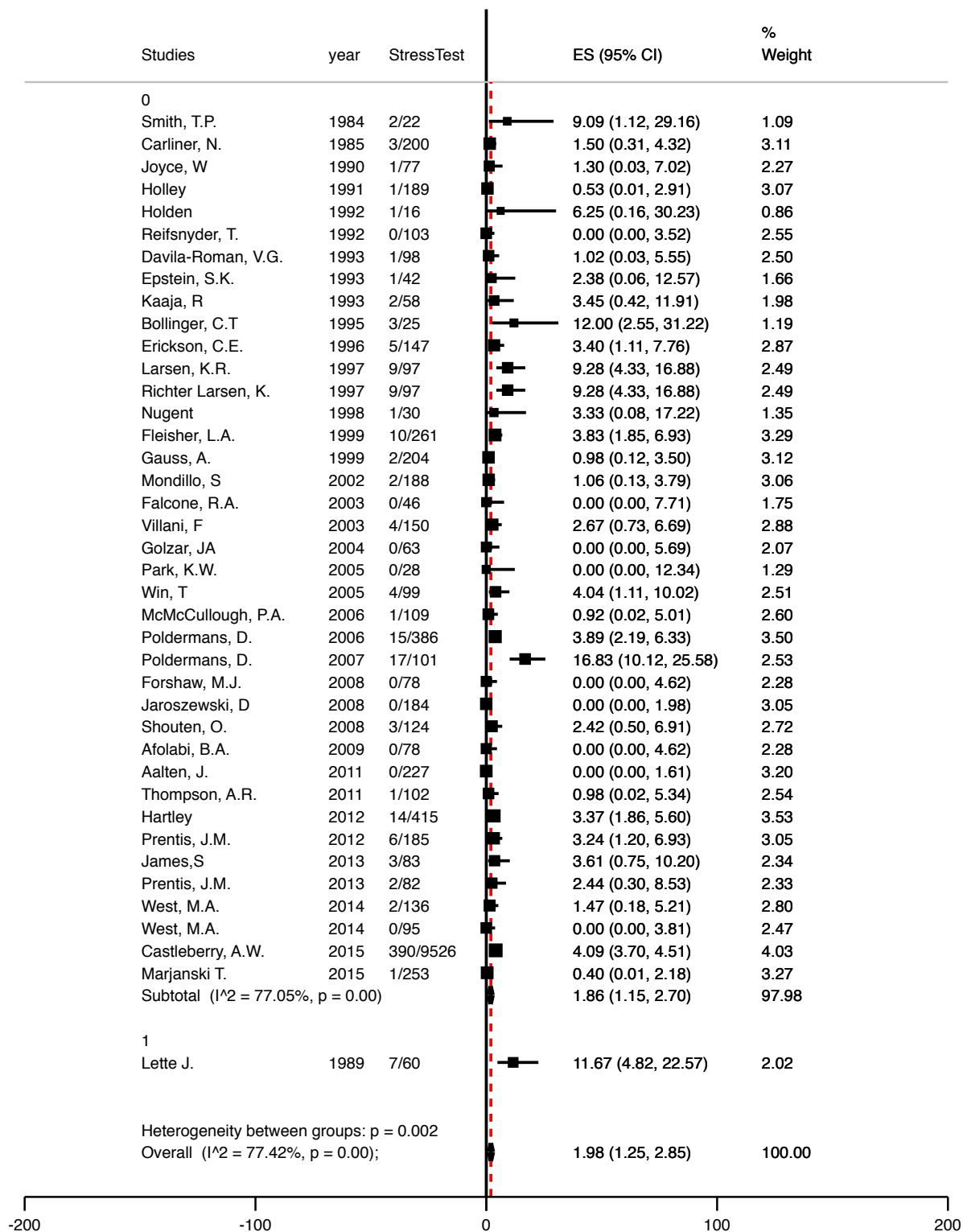

Supplement: S17 Fig — (PDF) [file pone.0219145.s017.pdf]

**Supplementary Figure 18: By type of surgery- gastric bypass**

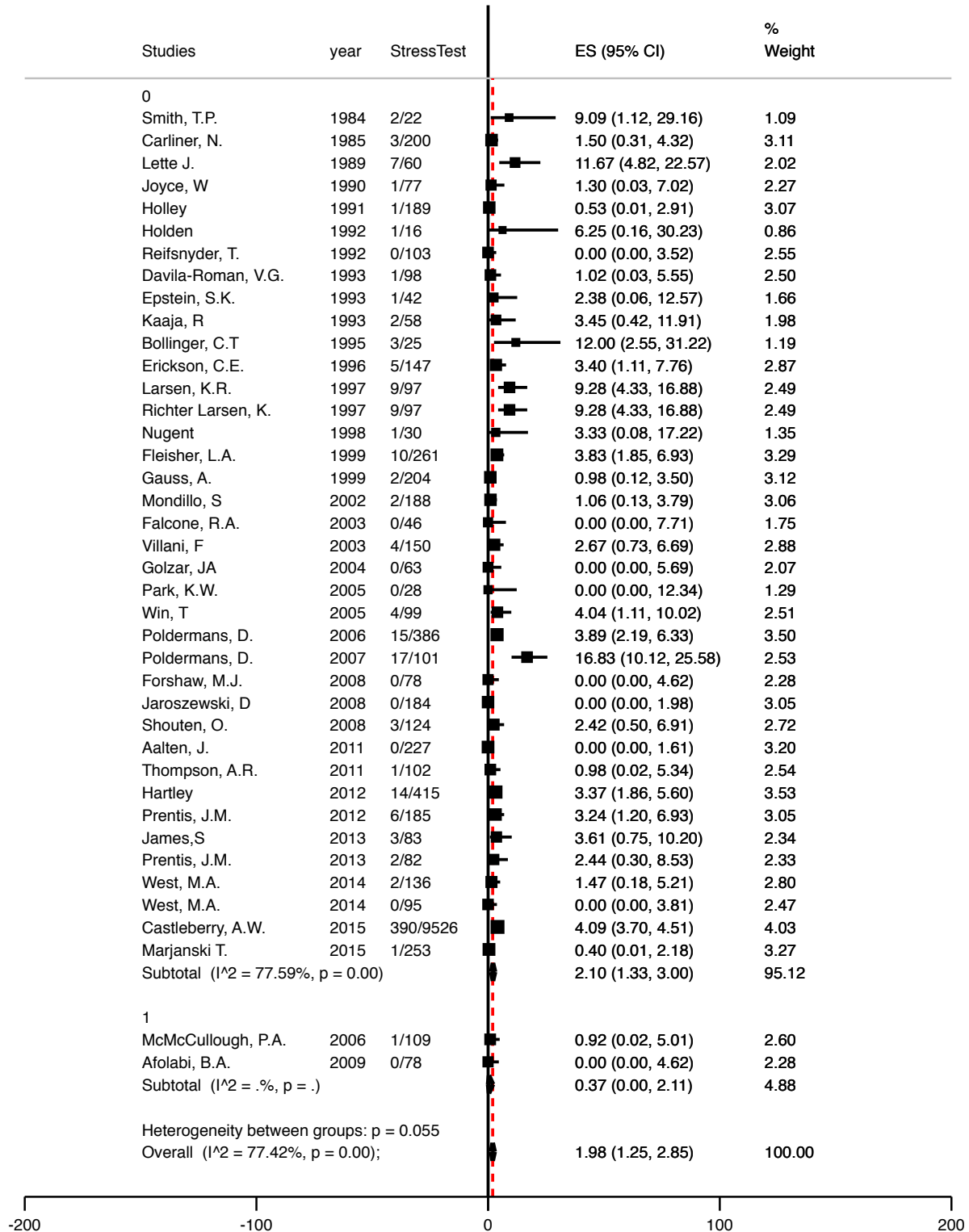

Supplement: S18 Fig — (PDF) [file pone.0219145.s018.pdf]

# Supplementary Figure 19: By type of surgery- other

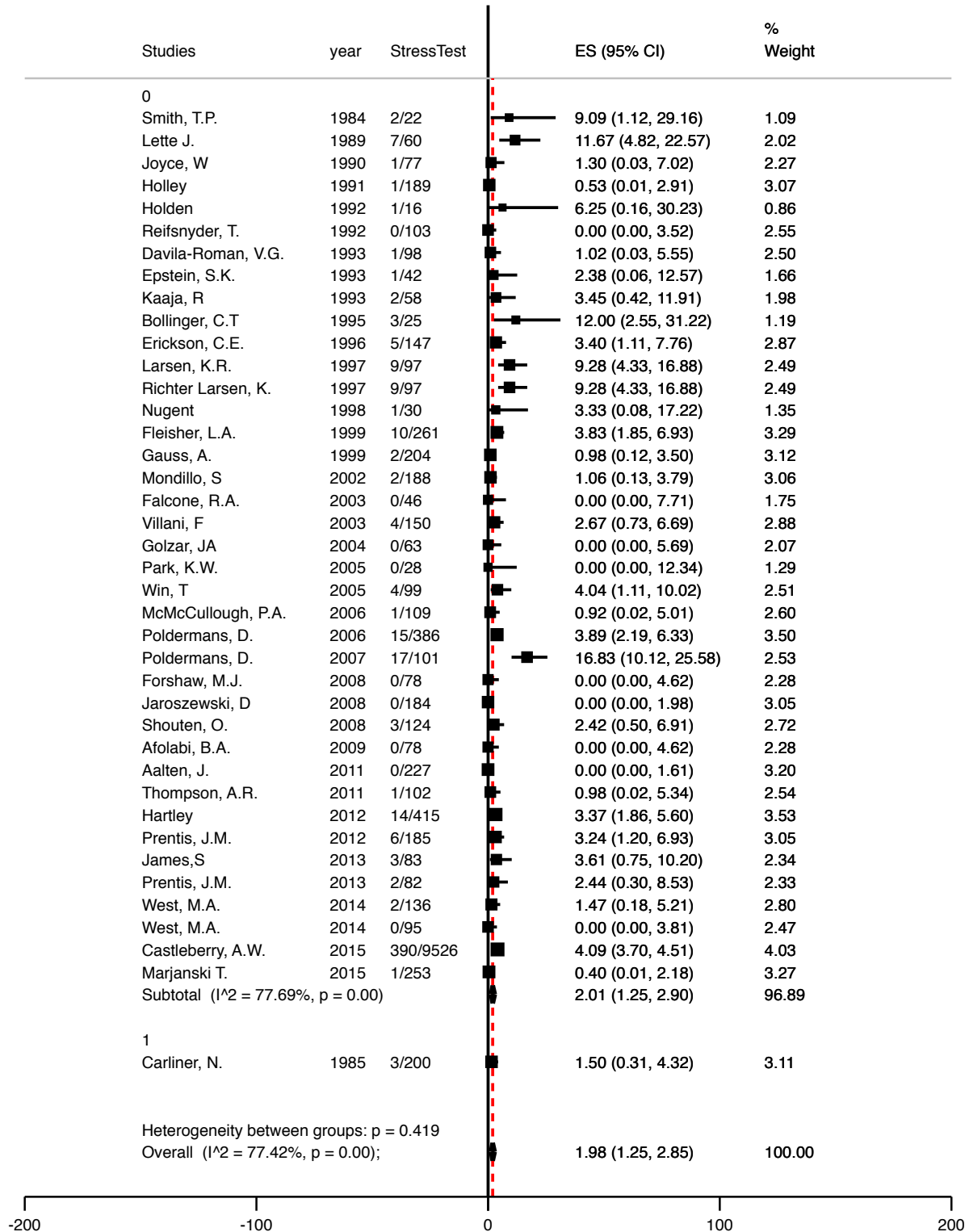

Supplement: S19 Fig — (PDF) [file pone.0219145.s019.pdf]
