# Supplementary material for: Pre-operative stress testing in the evaluation of patients undergoing non-cardiac surgery: A systematic review and meta-analysis
Source: PLoS One. 2019 Jul 11;14(7):e0219145. doi: 10.1371/journal.pone.0219145 (PMC6622497; doi:10.1371/journal.pone.0219145)
Supplement: S20 Fig — Eggers test = 0.17. (PDF) [file pone.0219145.s020.pdf]

Supplementary Figure 20: Contour funnel plot to highlight the effect estimate and standard error of 30-day mortality related to stress test in studies included in this meta-analysis, N=40 studies

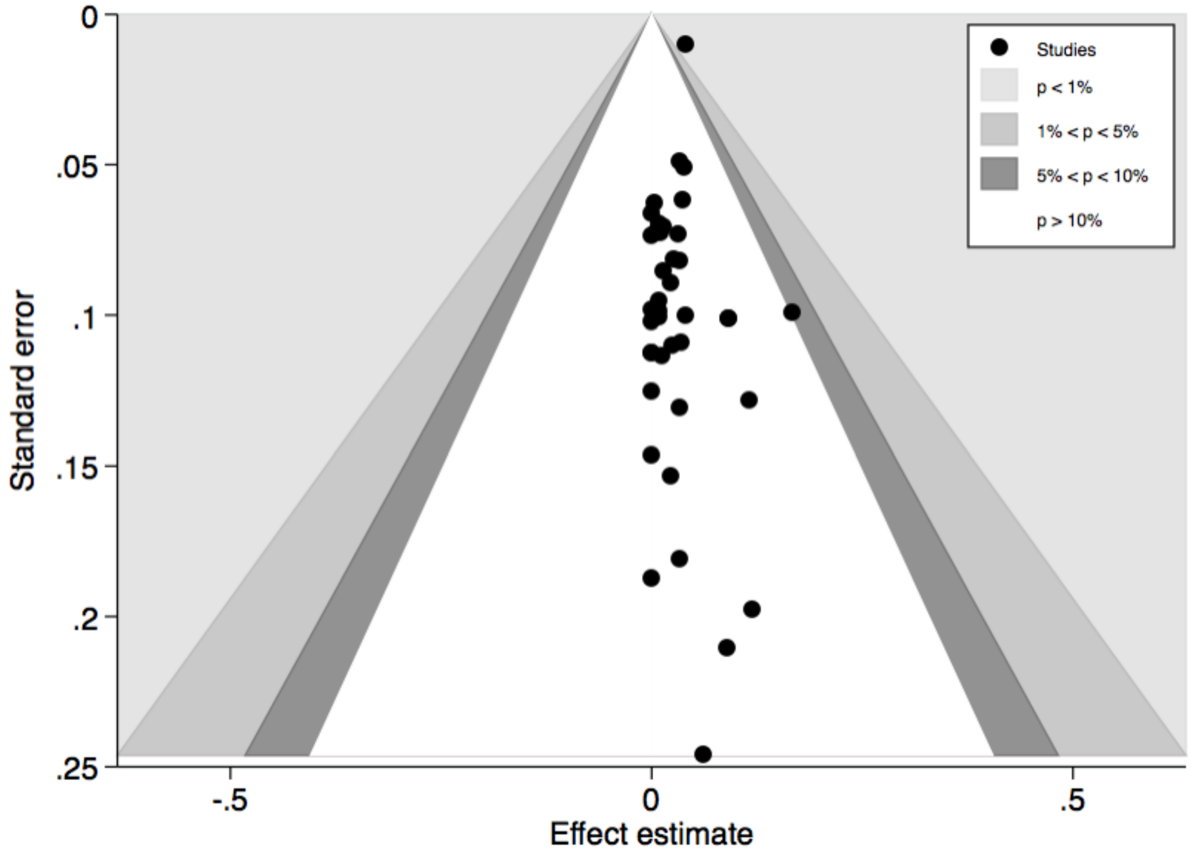

Eggers test=0.17
