# Supplementary material for: Pre-operative stress testing in the evaluation of patients undergoing non-cardiac surgery: A systematic review and meta-analysis
Source: PLoS One. 2019 Jul 11;14(7):e0219145. doi: 10.1371/journal.pone.0219145 (PMC6622497; doi:10.1371/journal.pone.0219145)
Supplement: S1 Appendix — (PDF) [file pone.0219145.s021.pdf]

S1 Appendix: Search criteria
Date of search: January 27nd 2016

Search strategy

Step	**MEDLINE** (http://ovidsp.ovid.com/autologin.html)	**Results**
1	stress test.mp. or exp *Exercise Test/	21375
2	exp *Preoperative Care/ or pre-operative.mp.	36738
3	exp *Preoperative Period/ or exp *Preoperative Care/ or preoperative.mp.	214998
4	2 or 3	229768
5	1 and 4	547
6	limit 5 to humans	525

EMBASE: (http://www.embase.com/home#/search:quickSearch/)

**Step**	**EMBASE**	
**1**	'stress test'/exp OR 'stress test' AND [embase]/lim	35,504
**2**	'preoperative' AND [embase]/lim	265709
**3**	'pre-operative' AND [embase]/lim	29359
**4**	#2 OR #3	283962
**5**	#1 AND #4 AND [embase]/lim	1353
**6**	#1 AND #4 AND [embase]/lim and [humans]/lim	1200

CENTRAL: (http://onlinelibrary.wiley.com/cochranelibrary/search/)

**Step**	**CENTRAL**	
**1**	MeSH descriptor: [Exercise Test] explode all trees	6522
**2**	Stress test	7840
**3**	MeSH descriptor: [Preoperative Care] explode all trees	5238
**4**	preoperative	18449
**5**	pre-operative	2475
**6**	#3 or #4 or #5	21452
**7**	#1 or #2	13448
**8**	#6 and #7	405

#hits per database:

MEDLINE: 525
EMBASE: 1200
CENTRAL: 405
Total: 2130 potentially relevant references identified with **at least** 323 duplicate references
