# Supplementary material for: Pre-operative stress testing in the evaluation of patients undergoing non-cardiac surgery: A systematic review and meta-analysis
Source: PLoS One. 2019 Jul 11;14(7):e0219145. doi: 10.1371/journal.pone.0219145 (PMC6622497; doi:10.1371/journal.pone.0219145)
Supplement: S3 Appendix — (PDF) [file pone.0219145.s023.pdf]

S3 Appendix: Results of phase 1 screening or abstract screening

Date: 08/12/2016

1. Unclear and clearly include were pooled and compared against all categories of exclusion
2. Comparison between HN and SI:
   Agreement= 90.4%
   <u>Kappa= 75.4%</u>
   Standard error= 0.02
   Z= 32.6; p<0.0001
3. Distribution
   Table 1: Basic distribution without unclear

	HN	SI		both
Clear include	244	202		201
Unclear	296	212		187
Missing	0	0		0
Exclusion				
1 = Ineligible population	467	621		429
2 = No intervention/comparator groups of interest	671	660		556
3 = Ineligible study design	23	32		14
4 = lack of adequate follow up	4	4		2
5 = Pediatric population	4	2		2
6 = Editorial, comment, review, conference proceeding theme	84	57		50
7 = Systematic review or meta-analysis	7	11		7
8 = Double entry of references (same abstract, in different journals)	7	6		5
9 = Protocol of RCT, quasi RCT or observational that fulfils inclusion	0	0		0
10 = not English language	0	0		0

4. Discordance

   Out of 1807, they have matched 1453.
   There is a discordance in 1807-1453= 354

5. Interpretation and Next steps

   Kappa is acceptable.
   Step 1: Reconcile the discordant 354.
   Step 2: Exclusion on 6 is inflated. Most of them will be excluded on 1. These have to be re-assessed.
   Step 3: There are too many unclear. These will have to be reassessed.

6. GB and HN re-assessed/cleaned up the endnote file as below

Clear include	163
Unclear	18

Missing	0
Exclusion	
1 = Ineligible population	485
2 = No intervention/comparator groups of interest	728
3 = Ineligible study design	39
4 = lack of adequate follow up	18
5 = Pediatric population	5
6 = not relevant outcomes	56
7 = Editorial, comment, review, conference proceeding theme	256
8 = Systematic review or meta-analysis	12
9 = Double entry of references (same abstract, in different journals)	17
10 = Protocol of RCT, quasi RCT or observational that fulfils inclusion	0
11 = not English language	10
