# Supplementary material for: Pre-operative stress testing in the evaluation of patients undergoing non-cardiac surgery: A systematic review and meta-analysis
Source: PLoS One. 2019 Jul 11;14(7):e0219145. doi: 10.1371/journal.pone.0219145 (PMC6622497; doi:10.1371/journal.pone.0219145)
Supplement: S4 Appendix — (PDF) [file pone.0219145.s024.pdf]

S4 Appendix: Phase 2 screening or full-text screening inclusion and exclusion criteria

c - Consider for **inclusion** during step 1 (all eligible non-cardiac surgery among adults

- c.1. peripheral vascular surgery or vascular surgery
- c.2. thoracic surgery
- c.3. abdominal surgery
- c.4. gynecological surgery
- c.5. urological surgery
- c.6. renal transplant surgery
- c.7. liver transplant surgery
- c.8 orthopedic surgery
- c.9 gastric bypass surgery
- c.10. abdominal aortic aneurysm surgery

u - **Unclear**, (choose if no abstract is available and the title is not enough to make a decision)

For **exclusion** use codes below, scoring in order of appearance (hierarchical exclusion):

1 = Adult Population NOT cardiac as described in c.1-c.10 (exclude all cardiac surgery and animal studies, cataract or eye surgery, ear, nose, throat surgery; dermatological surgery). Exclude on 1 if population is pediatric.

2 = neither of the 12 interventions/comparators

1. exercise tolerance test
2. pharmacological nuclear stress test
3. adenosine nuclear stress test
4. persantine nuclear stress test
5. dobutamine nuclear stress test
6. exercise nuclear stress test
7. exercise echocardiogram stress test
8. dobutamine echocardiogram stress test
9. cardiopulmonary stress test

10 metabolic stress test

11. stress test with gas exchange analysis

12 six-minute walk test

3 = Not RCT, not quasi-RCT, not observational study, not case-control study (case series satisfying c.1-c.10 of sample size <15 to be indicated as exclusion 2)

4 = If RCT, quasi RCT, observational, case-control (no follow up)

5= not the relevant outcomes.

6 = Editorial, comment, review, conference proceeding theme

7 = Systematic review or meta-analysis

8 = Double entry of references (same full text with or without minor differences, in different journals) (multiple reports on the same trial or observational cohort does not qualify as double entry)

9 = Protocol of RCT, quasi RCT, observational or case-control that fulfils inclusion

10 = not English
