# Supplementary material for: Pre-operative stress testing in the evaluation of patients undergoing non-cardiac surgery: A systematic review and meta-analysis
Source: PLoS One. 2019 Jul 11;14(7):e0219145. doi: 10.1371/journal.pone.0219145 (PMC6622497; doi:10.1371/journal.pone.0219145)
Supplement: S5 Appendix — (PDF) [file pone.0219145.s025.pdf]

S5 Appendix: Newcastle Ottawa Quality Assessment Scale

CODING MANUAL FOR COHORT STUDIES

SELECTION

1) Representativeness of the Exposed Cohort

Item is assessing the representativeness of exposed individuals in the community, not the representativeness of the sample of women from some general population.

Allocation of stars as per rating sheet

2) Selection of the Non-Exposed Cohort

Allocation of stars as per rating sheet

3) Ascertainment of Exposure

Allocation of stars as per rating sheet

4) Demonstration That Outcome of Interest Was Not Present at Start of Study

In the case of mortality studies, outcome of interest is still the presence of a disease/ incident, rather than death. That is to say that a statement of no history of disease or incident earns a star.

COMPARABILITY

1) Comparability of Cohorts on the Basis of the Design or Analysis

A maximum of 2 stars can be allotted in this category
Either exposed and non-exposed individuals must be matched in the design and/or confounders must be adjusted for in the analysis. Statements of no differences between groups or that differences were not statistically significant are not sufficient for establishing comparability. Note: If the relative risk for the exposure of interest is adjusted for the confounders listed, then the groups will be considered to be comparable on each variable used in the adjustment.
There may be multiple ratings for this item for different categories of exposure (e.g. ever vs. never, current vs. previous or never)

OUTCOME

1) Assessment of Outcome

For some outcomes (e.g. fractured hip), reference to the medical record is sufficient to satisfy the requirement for confirmation of the fracture. This would not be adequate for vertebral fracture outcomes where reference to x-rays would be required.

a) Independent or blind assessment stated in the paper, or confirmation of the outcome by reference to secure records (x-rays, medical records, etc.) ☆
b) Record linkage (e.g. identified through ICD codes on database records) ☆
c) Self-report (i.e. no reference to original medical records or x-rays to confirm the outcome)
d) No description.

2) Was Follow-Up Long Enough for Outcomes to Occur

An acceptable length of time should be decided before quality assessment begins (e.g. 5 yrs. for exposure to breast implants)

3) Adequacy of Follow Up of Cohorts

This item assesses the follow-up of the exposed and non-exposed cohorts to ensure that losses are not related to either the exposure or the outcome.

Allocation of stars as per rating sheet
